# Design and Development of an Intelligent Clinical Decision Support System Applied to the Evaluation of Breast Cancer Risk

**DOI:** 10.3390/jpm12020169

**Published:** 2022-01-27

**Authors:** Manuel Casal-Guisande, Alberto Comesaña-Campos, Inês Dutra, Jorge Cerqueiro-Pequeño, José-Benito Bouza-Rodríguez

**Affiliations:** 1 Department of Design in Engineering, University of Vigo, 36208 Vigo, Spain; jcerquei@uvigo.es (J.C.-P.); jbouza@uvigo.es (J.-B.B.-R.); 2 Department of Computer Sciences, Faculty of Sciences, University of Porto, 4169-007 Porto, Portugal; ines@dcc.fc.up.pt; 3 Center for Health Technologies and Information Systems Research–CINTESIS, Faculty of Medicine, University of Porto, 4200-450 Porto, Portugal

**Keywords:** breast cancer, expert systems, exploratory factorial analysis, data augmentation, machine learning, medical algorithm, clinical decision support system, design science research

## Abstract

Breast cancer is currently one of the main causes of death and tumoral diseases in women. Even if early diagnosis processes have evolved in the last years thanks to the popularization of mammogram tests, nowadays, it is still a challenge to have available reliable diagnosis systems that are exempt of variability in their interpretation. To this end, in this work, the design and development of an intelligent clinical decision support system to be used in the preventive diagnosis of breast cancer is presented, aiming both to improve the accuracy in the evaluation and to reduce its uncertainty. Through the integration of expert systems (based on Mamdani-type fuzzy-logic inference engines) deployed in cascade, exploratory factorial analysis, data augmentation approaches, and classification algorithms such as k-neighbors and bagged trees, the system is able to learn and to interpret the patient’s medical-healthcare data, generating an alert level associated to the danger she has of suffering from cancer. For the system’s initial performance tests, a software implementation of it has been built that was used in the diagnosis of a series of patients contained into a 130-cases database provided by the School of Medicine and Public Health of the University of Wisconsin-Madison, which has been also used to create the knowledge base. The obtained results, characterized as areas under the ROC curves of 0.95–0.97 and high success rates, highlight the huge diagnosis and preventive potential of the developed system, and they allow forecasting, even when a detailed and contrasted validation is still pending, its relevance and applicability within the clinical field.

## 1. Introduction

Breast cancer is, after lung cancer, the second main cause of death by tumoral diseases in women as well as one of the more frequently diagnosed malignant cancer types [1,2]. As a result of the high mortality and incidence rates of this type of cancer, since the last century, a strong effort has been made aimed toward the early detection of potential breast cancer cases before potential warning signs might appear in the patient. In this sense, it is common practice to perform population mammogram screenings on women that present higher-risk patterns such as age or ethnic group. In these last years, different studies were carried out that assess the efficiency and convenience of performing mammogram-based screenings [3,4,5,6,7,8,9,10], which generally conclude that they are a significant help to reduce the mortality associated to breast cancer, even if they might show a certain trend toward over-diagnosing [10]. As the main goal of mammogram-based screenings is preventive, by the early identification of patients at risk that are then derived to more specific tests, a significant reduction in cancer incidence is achieved [3,4,10]. Thus, it is reasonable to state that the efficiency in the interpretation of mammogram images is key in the detection, diagnosis, and potential treatment of breast cancer cases.

Mammogram images are usually interpreted by medical professionals, radiology experts, and oncologists that work together applying different criteria, analyzing the images, and trying to reduce the indetermination in the assessment. However, depending on the experience and training of the professionals in charge of the interpretation of the mammography images, the results obtained might show a certain degree of subjective variability [11,12,13]. This means that in some cases, it is necessary to carry out additional tests, which could be unnecessary in healthy patients to whom, therefore, harmful physical, mental, social, and economic implications could be avoided [14,15,16,17]. However, even if that might involve certain discomfort to the patient, the most controversial aspect derived from the indetermination in the diagnosis appears when it is concluded that a patient does not show a cancer case when in fact she does suffer from the disease, causing indefectibly a terrible delay in the detection of the pathology. It is relevant to indicate that the diagnosis methods based on medical images, such as mammography, echography, or magnetic resonance, are used as well as a complement in the confirmation diagnosis of breast cancer cases in women showing any characteristic sign or symptom, even if it is necessary to point out that at this moment there are not published studies, indicating that the absence of this implies the absence of breast cancer in the patient [18].

Within this healthcare context, which is common in countries having a centralized healthcare system, and in light of all those matters previously commented, in this field, it is key to have tools available that allow providing support to the difficult process of evaluating and diagnosing breast cancer, trying to minimize as much as possible its subjective variability, which is translated into medical terms as false positive and false negative diagnosis cases. Following this line, in the last years, many diverse methodologies and systems have been presented, aiming to provide support to clinical decision processes focusing on the task of helping the professionals in the arduous diagnosis task [19,20,21,22,23,24,25,26,27,28,29,30] (this to all practical effects considered as a decision) about the presence and evolution of different cancer types [31,32,33,34,35,36,37,38,39,40,41,42].

In this work, the design and development of a new intelligent clinical decision-support system is presented that provides support in the diagnosis process of breast cancer cases. Starting with the information and the characteristics extracted by the medical professionals from the interpretation of the mammogram images, as well as considering other data of interest about the patient (such as her clinical history), the system provides a quantitative index value of the hazard associated to the potential presence of cancer, from which interpretation it will be possible to determine the diagnosis and the evolution of the treatment. Thus, according to the hazard estimation, some additional tests (other additional image tests, biopsy, blood tests, etc.) might be recommended which, in case of a high hazard level, will confirm the presence of a cancer case or else will establish a series of routine examinations to continue monitoring the patient’s health status. To this end, and aiming to build a support system having higher diagnosis objectivity and lower diagnosis uncertainty levels, the use of an intelligent system is proposed that integrates symbolic artificial intelligence models, represented by the use of expert systems, and statistical inference computational models [43,44], through the use of classification non-parametric inference algorithms that are commonly used in Machine Learning. Aiming to properly explain and specify the operation of the system, Section S1 of the Supplementary Materials extends the previous description.

This work is organized and developed across four sections. In the current Section 1, its context is defined, identifying its advances and limitations within the field of study, as well as briefly enumerating its objectives. In Section 2, a conceptual description of the design and the definition of the Clinical Decision Support System is given, discriminating its different involved stages and events. After that, all the details required to understand the operation of the system are implemented and described. In Section 3, the results obtained from the case study where the system is tested are shown and analyzed. Finally, in Section 4, the presented system is discussed, and a series of critical conclusions about its usefulness and viability are posed.

## 2. Materials and Methods

### 2.1. Definition of the System

#### 2.1.1. Prior Considerations

In this work, a new clinical decision support intelligent system is proposed and presented, which is applied to the evaluation of the danger associated to the presence of breast cancer on a patient. The system allows generating a set of alerts, which help in the clinical diagnosis and evaluation processes.

The general theoretical framework on which this work is developed is the information systems context [45,46]. The developed system is contextualized as an intelligent decision system, integrated itself into information systems and technologies that make possible guaranteeing the data flow that is necessary for its operation. A large part of the success of decision support systems lies in the validity and processing of data, which facilitate the decision-making process itself, in particular regarding the processing of information. However, information processing made with the support of statistical tools having different scopes is not always enough when the decision incorporates criteria, circumstances, or perspectives that involve complex relationships among data. The incorporation of the ‘intelligent’ characteristic to the ‘decision support system’ term aims to solve that difficulty by complementing the processing of data with inferential models, deductive logics, and predictive algorithms that allow not only to search for relationships among data but also existing trends, causes, and consequences. The main challenge lies in appropriately combining and complementing all those tools, statistical, inferential, and predictive, to achieve a decision system having low uncertainty and outstanding accuracy. The tools used in the developed system are described in more detail in the Supplementary Materials to this work. It is recommended to see Sections S2.1 and S2.2 to better contextualize the tools composing the system. In the same way, the compliance of the system design with the guidelines proposed by Hevner et al. [45,46] can be found in Section S3 of those Supplementary Materials.

#### 2.1.2. Database Usage

A dataset containing data about 130 patients, collected from January 2006 through December 2011 at the School of Medicine and Public Health of the University of Wisconsin-Madison, will be used in this work for the definition and testing of the proposed system. After the surgical confirmation, 21 of the cases in said dataset resulted to be positive and 109 were negative. Demographic and medical history data (age, family history, patient’s history prior to cancer, etc.), BI-RADS© (Breast Imaging Reporting and Data System [47]) descriptors, as well as procedural information related to the performed biopsy and its results (type of needle and processes used, detected pathologies, etc.), together with assessments by several experts, were included by each one of those cases. All that allows to have available a collection of data that is structured and categorized into two classes or labels: patients that are a confirmed breast cancer case and patients without breast cancer evidence. As a summary, in Table 1, the main descriptors of the dataset used can be found.

#### 2.1.3. Conceptual Design and Description of the System

According to the characteristics of the developed system, the main concepts determining its design are referred both to the symbolic models represented by expert systems that will govern knowledge interpretation and to numerical statistical models represented by the data exploratory analysis and the statistical inference of the algorithms related to the classification and risk prediction processes. An assessment on the usefulness and fundamentals of these later tools can be found in Section S2.2 of the Supplementary Materials. Both approaches allow to control and manage uncertainty, which may be epistemological (derived for example from the mathematical model of from phenomenological aspects) or random [44,48]. All that is combined by means of a clinical decision support system applied to the breast cancer diagnosis process, especially in those dubious cases where the processionals are not sure about the decision. The ‘intelligent’ label for this decision support system is based on the combined use of artificial intelligence models that extend the traditional capabilities of these systems to incorporate reasoning, learning, and deductive logical inference capabilities.

**Figure 1:**
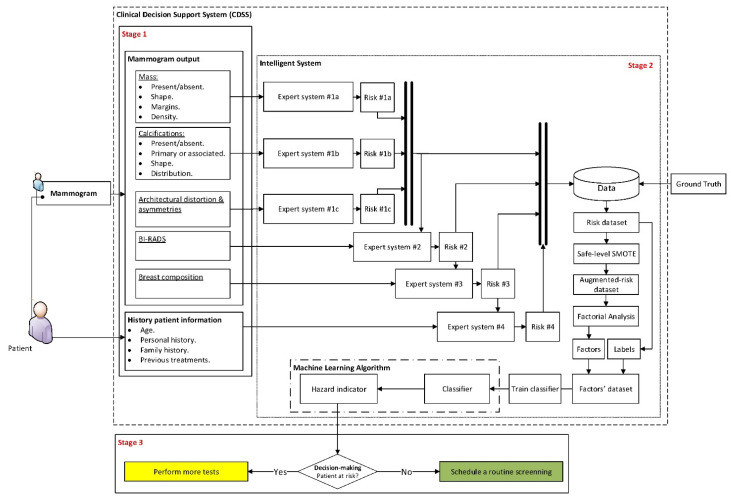
Diagram of the Clinical Decision Support System. This diagram shows the different flows of information across the three system stages. Stage 1 collects and groups the initial data, Stage 2 performs the data analysis and the inference of results, and finally, Stage 3 determines the decision of the system.

Figure 1 shows the diagram of the proposed Clinical Decision Support System, which will be described next.

##### Stage 1: Compilation of Characteristics and Other Information of Interest, and Expert Interpretation

The first stage in the proposed intelligent system is focused on the compilation of information. This may be split into two large groups: on the one hand, those that are related with the patient’s history (named ‘History patient information’ in Figure 1), and on the other hand, those that are associated to the extraction and interpretation of the different characteristics that are present in the image diagnosis test, in this case the mammogram. Such characteristics are based in prior annotations performed by the expert medical/healthcare staff (named ‘Mammogram output’ in Figure 1).

The group of data associated to the patient’s history may be seen in Table 2.

On the other hand, the dataset from the mammogram contains the following information in Table 3, of which a more detailed explanation is given in Section S2.3.1 of the Supplementary Materials.

##### Stage 2: Data Processing and Interpretation

Once the information has been collected and structured, it is time to carry out its analysis by the intelligent system.

**Figure 2:**
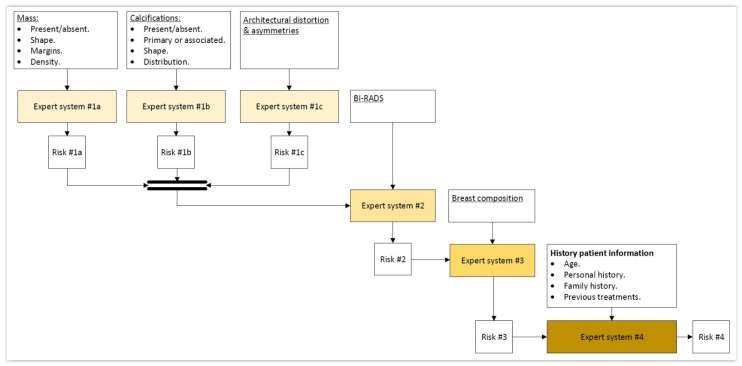
Detail of the expert systems cascade.

In the first place, the different previously shared and compartmented and commented information groups, shown in Figure 1, are processed by means of a series of expert systems working in cascade, using Mamdani-type fuzzy-logic inference engines [49,50,51,52]. Each one of these systems will produce an output associated to the risk of suffering cancer, and they will be individually related with all and each one of the previously commented information groups, as can be seen in the detail of Figure 1 shown in Figure 2.

The cascaded design of the expert systems obeys the need to have available a risk evaluation according to different criteria. Even if it is true that the conjoint assessment by all of them, listed in the previous section, makes up the final multi-criteria decision at the time of analyzing and incorporating the knowledge and expertise of this type of diagnosis; in this case, it is more appropriate to compartment the information. Not only the sizing of the information imperfection is achieved, but the rules on which the knowledge is modeled may be more precise and accurate. However, the multi-criteria assessment cannot be abandoned, because of which the output of an expert system is fuzzified before being used as input for the next one, thus achieving a progressive consideration of the influence of all criteria on the cascade’s end. The control of uncertainty is not associated to a probability value but to a membership value, with the Mamdani mechanism (on which the cascaded expert systems are based) being in charge of its management.

Once the risk outputs are obtained, each chain of them is labeled according to two main classes: the presence or absence of cancer in the studied patient. This labeling process is subsequent to the generation of risks in the cascade from the data initially collected and just prior to the supervised training process. The starting data will be the risks obtained after being structured and stored into a database, together with their corresponding labels. If this created dataset had an asymmetric trend and it was unjustifiably unbalanced, then the database should be processed by means of normalization and over-sampling procedures using Z-score and Safe-Level SMOTE [53,54,55], respectively. It is worth mentioning that the number of initial data will depend on the initial dataset and, alternatively, on the number of iterations made with the system after it was started up.

Finally, taking the final dataset and applying exploratory factorial analysis techniques, it is possible to determine a series of subjacent correlations existing in the data, considering the covariance patterns of the risks themselves, thus obtaining a collection of latent factors that group together and represent the different previously obtained risks. These factors will be used as assessment criteria by the classification algorithm. In this way, after obtaining the values of the different factors for each one of the patient cases and taking into account the previously mentioned labels, it is possible to train a Machine Learning non-parametrical classification algorithm, which after being set in operation would allow classifying the new patients.

It is relevant to mention that it will be necessary to have available an initial starting dataset already classified according to the main labels or classes of ‘cancer’ and ‘non-cancer’. This is not only essential for the generation of the rules but also fundamental in order to later create the algorithm training dataset.

##### Stage 3: Generation of Alerts & Decision-Making

A metric will be obtained as an output of the Machine Learning algorithm that is understood as the danger of suffering breast cancer, which needs to be interpreted taking into account the patient’s history, establishing a series of thresholds that will allow providing different indications. In this work, some indications or recommendations are proposed as shown next, but their modification and adaptation is a characteristic of the presented system in itself:

- Healthy patient → Schedule a routine revision.
- Dubious patient → Consider either to perform more tests or to schedule a new consultation after a specific time period.
- Cancer patient → Perform confirmatory diagnosis tests.

### 2.2. Implementation of the System

The system presented in Section 2.1 encompasses different stages that go from the compilation of information (Compilation of characteristics and other information of interest and expert interpretation), by way of its processing (Data processing and interpretation), ending in the elaboration of conclusions (Generation of alerts and decision making). The implementation of the system commented in this work is only circumscribed to the *Processing and interpretation of data* and the *Generation of alerts and decision-making stages*, which will be presented in detail. This is because the previous stages are related with the acquisition of information and its pre-processing, both questions being alien to the development of the software artifact, and that will be conducted based on the already described expertise and recommendations. Thus, the description of the implementation of the developed software artifact will be addressed next, explaining how the information is processed across the different screens of the user’s graphical interface.

The MATLAB^©^ (R2021a, MathWorks©, Natick, MA, USA) software environment has been used for the elaboration of the software artifact, employing the App Designer module for the development of a fully functional graphical interface. The inference systems have been implemented using the Fuzzy Logic Toolbox [56]. Developments made on MATLAB‘s own platform, distributed through the MathWorks^©^ File Exchange portal, were used for the implementation of Safe-Level SMOTE and exploratory factorial analysis, being available in the links of references [57,58], respectively.

**Figure 3:**
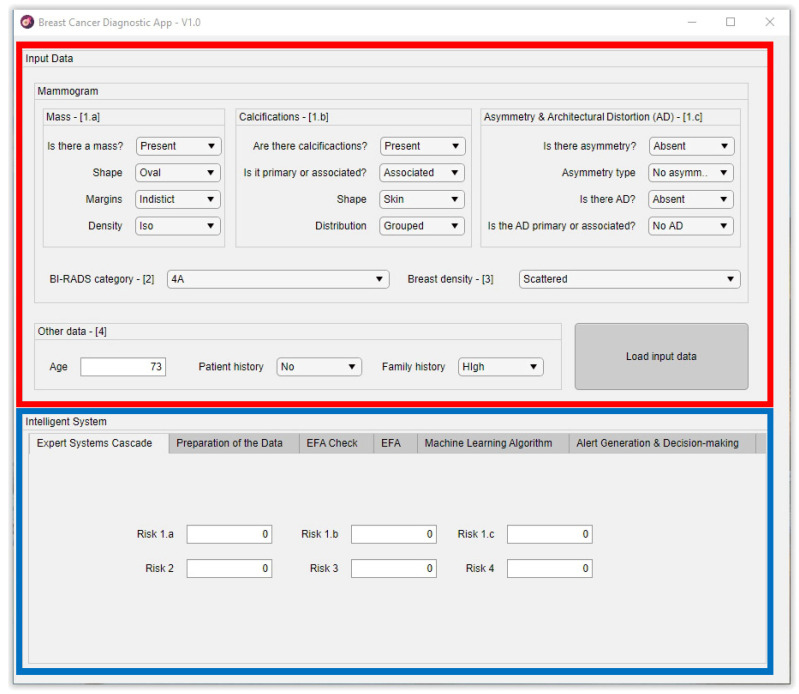
Screen capture of the application’s initial screen. [1.a] refers to the inputs of the Expert system #1a shown in Figure 1 and Figure 2; [1.b] refers to the inputs of the Expert system #1b shown in Figure 1 and Figure 2; [1.c] refers to the inputs of the Expert system #1c shown Figure 1 and Figure 2; [2] refers to the inputs of the Expert system #2 shown in Figure 1 and Figure 2; [3] refers to the inputs of the Expert system #3 shown in Figure 1 and Figure 2; [4] refers to the inputs of the Expert system #4 shown in Figure 1 and Figure 2.

Figure 3 shows a representative image of the main screen of the developed software artifact, differentiating two zones on it. The red frame marks the zone in the application used to input the starting data, which is enumerated in Section 2.1.3 in the *Compilation of characteristics and other information of interest, and expert interpretation* point. On the other hand, the blue frame indicates the zone associated to the intelligent system. The first four tabs correspond to that what was described in the *Data processing and interpretation* point in Section 2.1.3, while the last one corresponds to the same Section’s *Generation of alerts and decision-making* point.

In order to complement the contents of this sub-section, the different interfaces of the developed software are shown in Section S4 of the Supplementary Materials.

#### 2.2.1. Cascaded Expert Systems

As previously mentioned, once the starting information has been collected and structured, it is then processed first by a block of expert systems working in cascade that use Mamdani-type fuzzy-logic inference engines [49,50,51,52]. It can be seen in Figure 1 that the cascade has four levels, which are detailed next:

- Level 1: In the upper area of the cascade, in its first level, the processing of the information associated to masses, calcifications, asymmetries, and architectural distortions is carried out by means of three expert systems working concurrently [27,28,29,30,59,60], thus obtaining in each case, after the defuzzification process, an index value that expresses the risk level of cancer presence associated to each one of the groups of characteristics extracted from the mammogram.
- Level 2: In the second level of the cascade, the expert system carries out the processing of all the risk index values determined in the first level, those associated to the different mammogram characteristics, that is, to masses, calcifications, asymmetries, and architectural distortion. All of them are processed as inputs to the only expert system from this level, together with the BI-RADS^©^ index established by the medical-healthcare experts. A cancer risk level associated to the BI-RADS^©^ index and to the previously determined first-level risk indices is obtained after a defuzzification process, as an output of the second level expert system. It can be observed that even if the distribution is cascaded, the fact of incorporating the expert systems’ outputs as inputs of the following ones allows progressively grouping all the effects together.
- Level 3: The processing of the second-level risk, together with the information associated to the breast composition is carried out in the third level by the expert system. After performing the defuzzification, a cancer risk value is obtained that is associated to the composition itself of the breast and to the second-level risk, which was in turn associated to a BI-RADS^©^ category and to the first-level risk indices.
- Level 4: In this last level of the cascade, the processing by the expert system is performed of the risk obtained in the third level together with the information related to the patient’s history. As happened in the previous cases, the risk obtained in this fourth level is associated to the patient’s history and to the risk obtained in the third level, which was in turn associated to the breast composition and to the second level risk, and this in turn was associated to the BI-RADS^©^ index and to the first-level risks.

The cascaded use of expert systems extends their capabilities for modeling complex systems such as the one addressed in this work. The proposed cascade model, besides being progressive and sequential, allows incorporating as inputs of the current level the outputs of its previous levels. The expert systems that are present at each level progressively incorporate the information related to the different criteria for assessing the breast cancer risk in a way that the last level encompasses an inference of all the criteria by means of the fuzzification of the output risk from the previous expert system, which in turn collects the influence of all the previous levels. In turn, all the risk outputs are produced by defuzzification and they represent, in each cascade level, a cancer risk biased to a specific collection of input variables. The processing of the risk levels as consequents and antecedents, represented using membership functions that are specific to each case, makes it possible to model the degrees of membership of the risk concept according to two different approaches. Even if, formally, these represent the same amount of information and their meaning is similar, its fuzzification must be different, as the logical constructs in which they will be used will also be so. Additionally, in the case of the determination of the inference system rules, the fact that the systems work in cascade facilitates their determination, as the number of antecedents to consider is smaller. It is also interesting to point out that the determination of the risks associated to the starting data makes it possible to guarantee the anonymized processing or each patient’s data.

As the data from the different patients are getting processed by the cascaded expert systems, the different previously introduced risk indicators are being generated, which will be stored and structured into a database.

##### Calculation

All the inference systems used as engines within the expert systems of the previously presented cascade have a similar operation: they are fuzzy logic-based and of the Mamdani type [49,50,51,52].

**Figure 4:**
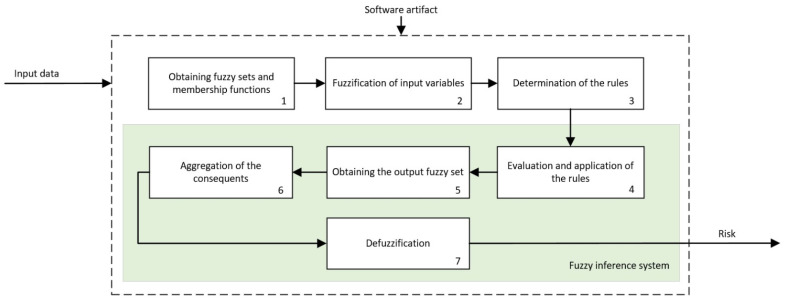
Schematic diagram of the inference systems.

Figure 4 shows the operation schematic diagram of these inference systems, which will be described next.

Following the schematic diagram shown in Figure 4, first, the membership functions for each one of the input variables are defined, which will allow determining the degree of membership as a specific value from the 0–1 range, with 0 indicating non-membership and 1 meaning the whole membership. As described in the previous section, the input variables are, in each expert system, assessment criteria associated to the interpretation of the mammograms as well as to the risk outputs of the previous expert systems that correspond only to the former level. Regarding the output variables, these will be the different risks associated to the inference of the corresponding input variables. The choice of the type of membership function is key, and it depends on the nature and characteristics of the represented variable as well as, in this case, on the place that it occupies within the logical construct. Thus, the risk values will be represented with different membership functions depending on whether they act as antecedents or as consequents of the deductive reasoning rules. This is so because, even if they represent the same information, their usefulness when reflecting deductive logical considerations of the *modus ponens* type is different if the variable is one of the true propositions or one of the implied ones. For example, if it is considered that proposition A is true and that A implies B, then B will also be true. The fuzzy nature of variable A, represented in its membership function, must accommodate all that qualitative interpretation of the truthfulness of A (or of any membership to a specific set). If the proposition A is in this case the cancer risk, when it acts as an antecedent, it will represent an assessment of that risk by means of qualitative language qualificators, and it will be represented by functions whose maximum membership level will have a certain variability, such as trapezoidal-shaped functions. However, if it is considered that B is true and that B implies A, then A is true. In this case, the certainty of A is a consequence of the deductive implication process and an always true premise according to the logical rules that guarantee the antecedent’s true value. The certainty of A, defined within a fuzzy set, will be collected in affirmation degrees that now could be pointwise and determined. Again, in the risk case, it will not be qualitatively evaluated, but it will be assigned a risk level. That is, if proposition A is assumed to be the risk and it acts as a consequent, such risk that indubitably exists might be represented by functions having pointwise maximum membership values.

Thus, in this case, a choice was made for using triangular membership functions for the variables derived from the interpretation and trapezoidal functions for those representing the precedent risks. In the first case, such a choice responds to the characteristics of the BI-RADS^©^ guide itself, and they allow maximizing the value of the function for a certain variable value. In the second case, as they are input variables, the trapezoidal function guarantees the existence of a certainty interval in the fuzzification. Once the membership degrees for the input variables are obtained, in the third stage, the production rules are established, both the declarative rules that are integrated from the knowledge base and the procedure rules themselves generated by the inference engine. Said rules will be of the ‘IF’… ‘AND’/’OR’ … ‘THEN’ … type, allowing representing the knowledge of the expert system by combining the different input variables and connecting them with the consequents. Actually, this knowledge is closely related to the medical/healthcare process and team that create the rules. Assuming an ideal behavior, the system will reason based on that knowledge, and it will be prone to be right or wrong as often as the human team that created it. The fourth stage deals with the evaluation and the implication of the rules. By using Mamdani-type inference, the ‘AND’ operator is related with the ‘intersection’ fuzzy operator which, when connecting different membership functions, returns the lower value membership degree, while the ‘OR’ operator, related with the ‘union’ operator, would return the larger membership degree. Once the antecedents have been evaluated, in the fifth stage, it obtains the consequents by applying the implication method, using in this case the ‘minimum’ one which truncates the consequent’s membership function, taking the value determined in the previous fuzzy operation. After that, in the sixth stage, the different consequents are aggregated by using the disjunctive approach [52] using the ‘maximum’ operator, providing a graphical output that equals the superposition of all the previously obtained consequents. From that superposition, an envelope is generated that determines the output set, which is later defuzzified in the seventh stage by using the centroid method [52], providing a numerical value that is associated to the risk of suffering cancer, as it has already been commented. The results obtained from each one of the cascade levels are shown in the *Expert Systems Cascade* tab of the developed application, which can be seen in the blue frame of Figure 3.

#### 2.2.2. Data Preparation: Normalization and Balancing

To use this intelligence system, trusting its predictive conclusions, it is necessary to carry out its setup/training, being essential for that to have available a categorized and coherent dataset. As previously mentioned, the first stage of the intelligent system is circumscribed to the calculation of the risks from the different starting information groups by means of a series of expert systems working in cascade. Said risks, obtained after the defuzzification process by the inference systems, are stored and structured into a database, together with the ‘cancer’ or ‘non-cancer’ label of the starting data.

It is common in medical datasets to observe cases in which the pathology to be diagnosed is a minority, what might cause the classification algorithms trained on that information to present undesired behaviors, forcing the production of high false-negative diagnosis rates. These unbalanced datasets, clearly asymmetrical, possess a biased and erratic behavior and must be processed before their use in production [61,62]. In this sense, to avoid such issues and to balance the dataset, in this work, a choice will be made toward the use of data-augmentation techniques on the dataset of the previously determined risks, more in particular using the Safe-Level SMOTE, having previously normalized the risks using the Z-score. The combination of these two techniques has proved, as reported in the literature, good results in medical datasets [55].

Equation (1) shows the expression for the *Z*-score calculation, with *x* being in this case the value of the specific risk index for each patient, and μ and σ being the mean value and the standard deviation for each risk index, respectively.

(1) $$Z-score=\frac{x-\mu }{\sigma }$$

On the other hand, to use the Safe-Level SMOTE, it is necessary to indicate to the algorithm how many synthetic data are to be generated and how many neighbors will be used in the algorithm operation. In this case, an amount of four neighbors will be used as the initial configuration of the algorithm.

After applying the normalization and data-augmentation techniques, the new values are stored together with those other already existing into the database, thus making available a balanced and statistically significant dataset.

#### 2.2.3. Determination of Latent Factors

Starting from the data that were normalized and augmented in the previous phase, in this stage, those subjacent latent factors that are present in the dataset are determined by using Exploratory Factorial Analysis (EFA) techniques, more specifically the Common Factor Analysis approach [63,64]. However, as a previous step to the application of EFA, it is necessary to verify the statistical assumptions that are recommended to ensure that the dimensionality reduction process is statistically significant. Said assumptions are often referred on the one hand to the nature of the data, assessing its normality and multi-collinearity as well as the presence of atypical data, and on the other hand to the number and interrelation of the data itself. The Shapiro–Wilk test, together with the estimation of the asymmetry indices, will be used for the normality case. The multi-collinearity will search for the presence of strong correlations among data that are harmful to the analysis; the Variance Inflation Factor (VIF) will be used for that. The Mahalanobis distance will be used for the detection of atypical or marginal data points. Finally, the number of data must be high enough, which is guaranteed by having a ratio of more than 10 data lines per risk. Regarding data interrelation, this must be guaranteed to exist, because of which Bartlett’s Test of Sphericity will be performed, together with the determination of the Kaiser–Mayer–Olikin (KMO) index.

It might be frequent that the used dataset does not verify all and each one of the recommended assumptions for carrying out EFA. For example, it might be that the normality criterion was not met in a strict way in each data column, obtaining however plausible values for the data interrelation statistics. Even if it would be advisable that all the assumptions were fulfilled, there are approaches within the EFA itself that could solve these small discrepancies and guarantee the statistical significance of the results. In fact, in the exploratory factorial analysis, the basic assumptions are more of a conceptual than of a statistical nature, because of which it is reasonable to omit the normality and linearity assumptions, accepting a reduction in the observed correlation [65,66,67]. Thus, it may be claimed that the exploratory analysis will have statistical significance only when the non-verified assumptions are accepted by the factors’ determination model.

Considering what has been previously shown, and always assuming that not all EFA criteria are met in full, then it would be necessary to use a method for the determination of the latent factors, or extraction method, that is robust before specific unfulfillments regarding those assumptions. There exist different extraction methods, the more common being Maximum Likelihood and Principal Axis Factoring [64]. The algorithm used in this work [58] applies Principal Axis Factoring. This approach presents certain advantages, as it does not require of a strict fulfillment of the assumptions by the starting data, such as the need that the data have a normal distribution, which will provide a higher versatility and the potential to be used with data that might not verify such conditions. Furthermore, its convergence is guaranteed by having available a large amount of data. Regarding the number of factors to be extracted, the algorithm uses Kaiser’s rule, in which only those factors having an eigenvalue value above 1 are extracted [64], checking that the indicated number, often used as the lower limit, is appropriate. Regarding rotation, this will be addressed aiming to improve the interpretation of the factorial loads matrix by means of an orthogonal approach, using Varimax in this case.

All the previously mentioned tests have been applied to the starting dataset with the goal of demonstrating and characterizing their usage. The process has been carried out on the developed software artifact, and it can be seen in more detail in Sections S4.1 and S4.2 of the Supplementary Materials. Thus, after performing the different tests, it was observed that the number of extracted factors was three. By analyzing these factors, it was possible to observe that they conjointly represent a variance, referred to the starting data, within the 0.75–0.82 range. After observing and interpreting the rotated loads matrix, it is possible to establish certain conclusions:

- For Factor 1, the risks having a larger influence are R_2_, R_3_, and R_4_, that is, the risks obtained in levels 2, 3, and 4 of the expert systems cascade. As a result of this, Factor 1 may be understood as that factor representing the BI-RADS^©^ index, representing the breast density and the patient’s history.
- For Factor 2, the risks presenting a larger influence are R_1a_ and R_1b_, two out of the three risks concurrently calculated by the expert systems in the first cascade levels. Thus, Factor 2 may be understood as that factor representing the masses and calcifications.
- For Factor 3, without any question, the predominant risk is R_1c_, which is one of the three risks calculated in the first system of the cascade. Therefore, Factor 3 may be understood as that factor associated to the asymmetries and the breast architectural distortion.

#### 2.2.4. The Machine Learning Algorithm

Once the different factors have been extracted starting from the risks in the augmented dataset and incorporating their labels (that is, whether the patient had or did not have cancer) and using exploratory factorial analysis approaches, a labeled dataset is available having a reduced dimension and statistical significance. As a result of that, it is possible to use it to train a statistical inference algorithm of the types commonly used in Machine Learning. By using a classification algorithm, it will be feasible to perform predictions for new data. It is clear that such an algorithm will be of a non-parametrical type according to the nature of the data itself, for which it will not be possible to a priori guarantee any of the requirements of parametrical statistics.

Aiming to explore in a simple and efficient way the different possibilities, different tests were carried out using MATLAB’s Classification Learner App [68], which allows training different learning algorithm types in a massive way.

To perform the validation of the different models, a choice was made for using cross-validation techniques, more specifically k-fold cross-validation, taking 5 cross-validation folds. Cross-validation is often used for the training and validation of Machine Learning algorithms. In essence, this technique splits the dataset into K sub-sets or ‘folds’ so that one of them is used to perform the algorithm validation while the other ones are used for its training. The process is iterated until all K sub-sets have been considered.

**Figure 5:**
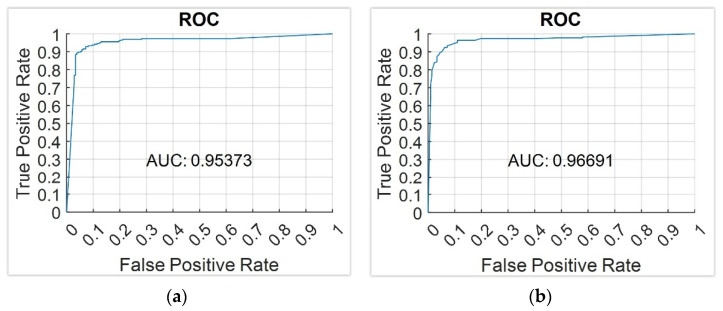
ROC curves for the best models. (**a**) Weighted KNN model; (**b**) Bagged Trees model.

Same as in the previous sub-section, and deriving the results obtained from it and the used dataset, the graphs of the ROC validation curves for the different models were used for the selection of the model. Among all the trained models, the best results were obtained with the Weighted KNN and Bagged Trees algorithms whose data are shown in Table 4. Figure 5 shows the ROC curves for the previously mentioned models, which are very similar to each other.

In line with that already explained, in the developed application, the user is given the choice of using any of these two models. However, in the future, and according to the different data and the observed particularities, any other algorithm could be used without any harmful effects. The software interface managing this process is explained in Section S4.3 of the Supplementary Materials.

#### 2.2.5. Generation of Alerts and Decision Making

Once the algorithm has been trained and the scores for the patient to be evaluated have been determined, then it is necessary to determine the patient status: that is, how it is recommended to proceed. In this work, in order to carry out the evaluation of the patient, the previously mentioned Hazard index will be chosen.

In this sense, prior to the evaluation of the Hazard index, it is necessary to establish the limit values that will determine the system statuses and therefore the different recommendations:

- Status 1 is associated to a healthy patient, recommending the medical/healthcare professionals to propose a routine revision. This status will be recommended when the Hazard index value is in the [0-Limit_1) range.
- Status 2 is associated to a dubious or indeterminate patient status. A revision will be proposed to be held after a specific time period to re-evaluate the patient status and to make new decisions. This status will be recommended when the Hazard index value is in the [Limit_1-Limit_2] range.
- Status 3 is associated to a patient with a noticeable trend to suffer of cancer, recommending the performance of more tests to confirm the diagnosis. This status is recommended when the Hazard index value is in the (Limit_2-100] range.

## 3. Results

In this section, the results derived from the concept testing (or case study) of the intelligent system presented in this work are shown. As its name suggests, this test does not intend to validate nor to benchmark the system but only aims to provide an example of its application and to determine its viability and its potential as a diagnosis complement. To that end, starting from the dataset that is available, a data line from it will be extracted, corresponding to a patient who will be the diagnostic target for the system. The remaining data will be used for training the algorithm and creating the declarative rules that make up the core of the expert system knowledge base. The reason for extracting a data line from the starting dataset itself lies in that, evidently, the diagnosis must be carried out on patients whose data have been collected in similar circumstances, using similar assessment criteria, and evaluated under the same parameters. Otherwise, the concept test would lack formal validity, as the system’s reasoning and predictive capability would be subject to specific circumstances, while the prediction would be validated against spurious data.

Considering that, next a short usage guide will be shown, starting from the available dataset, and aiming to categorize the medical case of a representative patient that was chosen from those available. It is evident that as explained before, that patient’s data were not present in the dataset used for the system training, being absolutely independent of any internal learning and validation process whatsoever.

### 3.1. Compilation of Characteristics and Other Information of Interest, and Expert Interpretation

As already mentioned, the presented system starts its operation with the data being input by the professional into the application. Table 5 shows a summary of the patient’s data to be analyzed in this case study. She is a 53-year old patient with a family history of low cancer risk without having previously debuted in cancer. After evaluating her mammogram, it is possible to observe the presence of associated calcifications, with a coarse heterogeneous shape and a segmental distribution. A focal asymmetry was also observed. It is relevant to highlight that this patient was diagnosed with cancer.

### 3.2. Data Processing and Interpretation

After inputting the data into the application, it is possible to proceed to its processing by the system.

First, the risks calculation is carried out in the cascaded expert systems, as previously mentioned in Section 2.2.1.

The risk value associated to the masses, R_1a_, shows a value of 0.01538, the risk value associated to the calcifications, R_1b_, shows a value of 39.97, the risk value associated to asymmetries and architectural distortion, R_1c_, shows a value of 70.03, and finally, the risk value associated to the BI-RADS© indicator and to the first-level risks, R_2_, shows a value of 89.98. On the other hand, the risk value associated to the breast density and to the risk values of the first and second levels, R_3_, shows a value of 80. Finally, the risk value associated to age, patient/family history, and to the first, second, and third levels, R_4_, shows a value of 89.97.

As it can be observed, in this case, the higher risk values are those associated to the architectural distortion and asymmetries, to the BI-RADS, to the breast density, and to the patient’s age/history, that is, the R_1C_, R_2_, R_3_, and R_4_ risks.

Once the risk values for the patient to be studied have been calculated, it is proceeded to load the training dataset, to perform their normalization, and to apply Safe-Level SMOTE (a clear asymmetry is observed in it), adding 189 samples for the ‘cancer’ class and 100 for the ‘non-cancer’ one, which makes the training dataset to have almost the same number of ‘cancer’ and ‘non-cancer’ classes, presenting more than 400 data lines. The normalization of the previously calculated risks for the patient to be studied is also performed.

Taking the augmented dataset, exploratory factorial analysis techniques are applied next after verifying its pertinence, as previously described in Section 2.2.3. In line with that already explained, three latent factors are extracted which, on this dataset, allow explaining 75.28% of the total variability.

The starting values (the risks) have been also mapped to the new factors space, using the Anderson–Rubin factorial scoring method both for the dataset and for the data of the patient to be analyzed.

Once this is done, it is proceeded to train the Machine Learning model, using Bagged Tree in this case, as described in Section 2.2.4. The scores for the patient’s data are also calculated, obtaining a Hazard index value of 66.67.

All those previous steps are shown as screen captures of the software interface in Section S5.2 of the Supplementary Materials.

### 3.3. Generation of Alerts and Decision Making

After the model has been trained and the scores for the patient to be studied have been calculated, it is possible to proceed to the generation of alerts and the decision-making process.

Taking into account the limit values of 60 and 65, which are established after analyzing the ROC curve, in this specific case, the system is facing a potential cancer case, in which it is recommended to the medical-healthcare professionals to perform more tests, starting from the least aggressive ones, that could help to make a diagnosis decision, determining whether it will be finally necessary to proceed to biopsy tests. Same as in the previous point, the corresponding screen captures of the software interface can be seen in Section S5.3 of the Supplementary Materials.

### 3.4. Interpretation of the Results

As explained at the beginning of this section, the case study developed as a concept test is not aimed to provide a full and contrasted validation of the presented diagnosis system but rather to show its use and assess its suitability for cancer prevention. In this line, from the analysis of the results, two perspectives may be extracted: one of them focused on those technical aspects that made it possible to contrast the combined use of the different inference techniques used and another one related to the clinical aspects that point to the diagnostic, predictive, and preventive potential of the system. Analyzing the specific results, AUC values of 0.95–0.97 were obtained in the training process by using cross-validation, which highlights the accuracy of the knowledge models used and the precision of the symbolic model. Additionally, the system produces a cancer risk value of 66.67% which, after being appropriately interpreted considering the established threshold values, matches the patient’s real condition. Considering that this behavior was obtained using a dataset containing only 129 patients’ data (it must be remembered that the case study patient was removed from the set), the clinical relevance of these outcomes results are promising, at least.

#### Expansion and Confirmation of the Results

With the goal of reasserting that interpretation, the patient to be diagnosed was replaced, and new examples were developed following an operative similar to that described in this section and extended in Section S5 of the Complementary Materials. It was chosen data from patients who on the one hand had a confirmed cancer diagnosis and on the other hand presented a symptom case whose assessment resulted to be particularly difficult to the medical/healthcare staff. The obtained results have been collected in Table 6, where the trend observed in the main case study can be seen and confirmed. Eleven diagnoses were carried out, where the system correctly categorized all the cancer patients (2) and identified seven patients who did not have cancer. The two remaining patients were labeled by the system as uncertain. The data associated to the first of them did not contain information on neither the family nor the clinical histories, and they had a 4B BI-RADS category, which is considered as a high-risk one (see Table S3 in the Supplementary Materials). The data on the second patient show a clear architecture distortion, as well as a 4C BI-RADS category, which is considered as a very high-risk one. Even if in both cases, the diagnosis finally indicated that cancer was not present, the system was not able to warn of the existing risk, considering the assigned BI-RADS category and the difficulty of interpreting the diagnosis signs, and only after the biopsy it was possible to discard the presence of the disease. With all that considered, it can be claimed that the system possesses an outstanding diagnosis success rate within the scope of all the posed examples, and that confirms its potential and relevance for its intensive use in clinical environments.

## 4. Discussion

In this work, a novel clinical intelligent decision support system has been introduced, whose key objective is to provide assistance to the breast cancer diagnosis process. As a result of the relevant incidence of this disease, it is nowadays more needed than ever to have available decision support tools that allow addressing difficult decisions, especially those related to the patients’ health and wellness. The presence of intelligent decision-complementary models, as well as the novelty that the system presented in this work involves, are discussed in depth in Section S6 of the Supplementary Materials, highlighting the differentiating character that is associated to the system’s internal architecture. This architecture incorporates into its design and development a collection of different knowledge models, integrating expert systems, exploratory factorial analysis (taking into account normalization and data augmentation), and statistical inference systems. Although they might be considered as independent, in this work, it has been proved that the combined use of all of them not only allows improving the decision-making process acting on the information form and contents but also optimizing its accuracy ratio, as it has been proved in the results derived from the analysis of the ROC curves. Taking, for example, the value of the area under the curve (AUC), values of 0.95–0.97 are obtained, which suggest really high diagnosis accuracy values, as a value of 1 would imply the maximum accuracy level. The interpretation of these results, beyond the quality of the dataset and the proved efficiency of the previously described inference learning models, is related to elements that are common to said models, such as knowledge representation and uncertainty management. Each one of those models will be commented next:

- Expert Systems: Expert systems are the paradigm of deductive symbolic reasoning applied to the artificial engineering field. In this work, their diversification and formalization fundamentals are implemented by means of cascaded information management. These systems diversify information, as they not only allow compartmentalizing it while keeping a common goal but also incorporate the definition of the declarative rules that model knowledge at each risk assessment stage. The generation of these rules on which the reasoning is made must be always supported on logical structures representing compiled and evaluated facts in similar circumstances and making use of a knowledge that is similar as well. There is an inherent dependence between who creates the rules and the way of reasoning of an expert system, and that implies assuming the presence of doubt or error in the process. Thus, the generation of such rules always involves accepting some level of uncertainty, because of which an interpretation of the membership function associated to the formalization of information was considered. Precisely, as the formalization is an inherent feature of expert systems, it is achieved by sharing the consequents of the four cascade levels. All of them represent the risk of suffering of cancer, modeled as a technical variable, and in turn, they incorporate that same variable in the inputs of the consequent expert system but already represented as a qualitative variable. This distinction lies in the own groundings of the deductive reasoning that, by means of the fuzzy logic-based inference engines, feeds and provides logics to each expert system. As it was already mentioned in Section 2.2.1, the consideration of antecedent or consequent of the same premise or variable in a declarative rule must affect its fuzzy nature, which is inevitably associated to the indetermination of its quantitative representation. This is the way in which the expert systems cascade manages uncertainty (considered in this work as both a metric of the measurement uncertainty and the indetermination of knowledge) by means of a system where, in a formal way, the descriptors representing the input and output variables are subject to first-order logical considerations, while delimiting the scope of the represented knowledge and expertise themselves. The control of uncertainty is carried out by reducing the complexity of the logical construct representing the knowledge, but in turn accumulating it once such variability has been progressively reduced in the earlier cascade steps. According to the literature review carried out by the authors, nowadays, no cascaded expert systems model can be found in it, and thus, this work involves a differential and novel contribution.
- Exploratory factorial analysis: It might be convenient not to mix exploratory analysis with statistical inference models. The former, according to the initial approaches by Tukey [69,70], extends the variance and typical deviation-based analysis by including a deeper analysis on the variables’ correlations with respect to the subjacent non-measurable that group the variables themselves together. In this work, the exploratory analysis is used to reduce the dimensionality of the labeled risks set obtained after applying the expert systems cascade as well as finding hidden and significant relationships among variables. When the risks are obtained, both within an experimental measurement approach and within an observational approach, the exploratory analysis is able to correlate the risks and to group them together into latent factors, which in turn they explain. The knowledge model is probabilistic in this case, and the information is grouped according to the covariance matrices and the rotations orthogonality assumptions. However, the exploratory analysis shows a changing dependence on the conditions of the starting data, which is why Common Factor Analysis was applied in this work. Nevertheless, that involves accepting a series of restrictions to be fulfilled by data, as described in Section 2.2.3. The acceptance of that limitation must be justified in the results and in a representation symbolic model that, even being ambiguous, is represented by the variables’ commonalities themselves. The covariance with respect to a factor not only represents an explicative dependence of the factor’s conceptual significance, but it implicitly represents a consequent explanation of the other factors, which is emphasized after the rotation. This means that the risks are grouped together according to latent factors, difficult to categorize but representing an inductive reasoning that is similar to the one that a medical-healthcare professional would develop in a consultation. Before the absence of accuracy, the multi-criteria decisions are supported by discussable quantifications on the degree of fulfillment of those decision criteria. By using EFA, the criteria are the factors, while the degree of fulfillment is set by the covariance of its explanatory variables. The uncertainty appears here delimited in probabilistic explanatory terms for the EFA application assumptions. That is, if EFA is comparable to a multi-criteria analysis, then guaranteeing its statistical significance means also tacitly guaranteeing a certain metric for accuracy in its recommendation. Therefore, EFA, fed with values where the qualitative risk has been already delimited, provides a numeric control on the imperfection of information. Thus, the justification of EFA’s fulfillment will be a necessary condition.
- Data normalization and augmentation: Together with EFA, the data normalization and augmentation have a single objective in this work: to guarantee the applicability and relevance of the search for factors. Even being a clearly artificial approach in the application of the decision system, its justification is based on its conceptual representation. To practical effects, over-sampling generates an artificial dataset by using a metric approach as a generator. Even though this may mask, and even bias, the data, that is not always the case for medical datasets focused on the use of factorial analysis. In a medical consultation, in any diagnosis process, it is reasonable to think that the dataset will be inclined, as the number of collected data increases, toward the presence of distributions that are normal and show a trend for a low collinearity. Similarly, in all random and non-biased data collection, the data will be uniformly distributed around its mean point, reaching typical deviation values nearing 1 and means nearing 0. In fact, that is why hierarchization modes such as the Ordered Weighted Average [71] are based on normal models for their classifications. As a result of all that, Safe-Level SMOTE does not unreasonably alter the data, but it generates a dataset fulfilling the EFA precepts and, in the case of having available a large dataset, it even would not be necessary.
- Statistical inference systems: The last of the approaches that has been incorporated into the system that is proposed in this article is related to statistical inference, that is, with reaching plausible conclusions from the obtained data that characterize a specific problem. On a broader scope, the statistical inference is used in Machine Learning by means of different families of regression and classification algorithms that allow, after a supervised training process that involves labeling data into specific classes, to obtain a prediction that is based in that training and its validation. In this work, the determination of the classification algorithm is not especially relevant, as it may be in general claimed that any approach used (decision trees, Naïve Bayes, support vector machines, etc.) could achieve relevant results. The reason for that lies in the data that feed, train, and validate the algorithm. Beyond its non-parametrical design, these algorithms possess trustworthy capabilities for finding relationships in datasets that are asymmetric or that have distributions that are far from normality. However, this versatility does not exclude that balanced and normally distributed datasets could not reach significant results such as those shown in this work. In any case, and in the same way that happened when creating the knowledge base declarative rules, the datasets used for training, validation, and forecasting must meet the premise of being obtained in similar circumstances and fulfilling equal diagnosis criteria so that their relevance in this clinical process is analogue. The training must be performed using data having a meaning and a significance identical to that from the data using in the forecasting, as the algorithm objective would become distorted otherwise. Additionally, same as for the EFA, the processing of uncertainty is carried out both from the epistemological and from the random approaches, considering ambiguity and interaction [48] with a probabilistic approach to it. The excellent results shown in the ROC curves support, precisely, that chain of events that has been previously described.

Finally, it might be worth considering the use of the system itself as a habitual diagnosis tool. As it has been already commented, the implementation of the software artifact has followed the guidelines by Hevner et al. [46], which determines that it might be incorporated without difficulties to production information systems such as those that are common in hospitals and national health systems. In the same way, regarding its use, the only interaction with the user takes place at the time of inputting the data for the patient to be assessed, as the prediction needs them to be completed. However, the elaboration of the knowledge base is a prior process that will require not only the participation of a medical-healthcare expert but also that of a multi-disciplinary and international team. The creation of declarative rules possesses an indetermination component that must necessarily be controlled in order to avoid the expert system producing results that are contrary to deductive logic. Even if, as mentioned before, the diversification and formalization of the information might help in this task, an efficient knowledge and expertise representation is needed from the supervision of the permanent symbolic system. This implies that despite the system’s design being aimed toward its almost-autonomous operation, it is required, especially in the first implementation stages, a constant revision of the symbolic (not as much of the numerical) models to guarantee an appropriate use reliability that is comparable to other simpler decision models. In this sense, simpler multi-criteria decision models, or even models incorporating expert systems or Machine Learning algorithms [27,28,34,35,39,40,41,42], reach success results that are similar to those presented in this article but always addressing problems with a lower complexity than breast cancer diagnosis has. However, it is necessary to highlight a key aspect: the presented system achieves the mentioned results with very few use iterations by combining different inference and analysis models together with rule modeling without a ready medical supervision. It is expected that as new relevant and homogeneous medical-healthcare assessments (multi-national and based on the system’s expertise) are available, the system will at least maintain its success rate. Additionally, and because of its architecture, in case it is deemed necessary, not only the classification system (already a user option) might be modified, but even the EFA model and/or the expert systems’ symbolic inference engine could be changed as well. All that invites thinking that the system has constituted itself as a very useful decision support tool that has, in particular, enormous possibilities for its future use. In this line, immediate effects such as the robustness of the exploratory analysis or the internal modification of the predictive algorithm are particular advances. Nevertheless, other improvements such as those related to the correct fuzzification of the input and output variables, the wider and more accurate knowledge representation by means of declarative rules, and even the improvement in data augmentation imply solutions with a larger scope and depth of study. Even so, as the next natural step, the system must be implemented for its use into a real production environment to be progressively optimized from all the data collected in this phase.

However, despite the observed and previously described potential, the system is still in an initial stage of its validation process. From a clinical viewpoint, our system still has shown neither a direct applicability nor enough reliability to support decisions as relevant as cancer diagnosis. The limitations associated to the case study are undeniable, and they open the door to new validation and benchmarking tests using larger datasets, with independent training and validation sets, and even utilizing new confirmatory datasets. Even so, because of the need that the data have a similar diagnosis environment regarding its collection circumstances and criteria, all the used data must be reviewed and compared to guarantee that their values can be compared and matched to each other. In the same way, it is clear that the generation of rules and the creation itself of the knowledge base results are easier when using labeled and structured datasets, which allows creating contrasted deductive logical rules. It seems reasonable to think that in real application environments, said circumstances will not always favor finding those casual relationships immediately, potentially implying a crossed influence among the diagnosis data, and besides that, those data might not be classified according to such order and homogeneity levels. As it has already been explained, despite all these undeniable limitations associated to its current development stage, the system is equipped with internal tools aimed to alleviate them. The search for causality may be reviewed and partially solved by means of the results of the expert systems cascade. By means of exploratory analysis, it is possible to reduce the data dimensionality and to identify existing crossed relationships among them. The lack of homogeneity is reduced by creating rules that point toward input data that are internationally recognized such as BI-RADS and the presence of non-classified data might be addressed by using data augmentation. In the same way, the system allows modifying the final classifier, with which a versatility and adaptability component is introduced that in many cases depends on the input data themselves. The design of the system includes all those components that anticipate many of the future limitations associated to the intensive clinical use of such system, and because of that, it is reasonable to think that after the future and forthcoming benchmarking and validation tests are carried out, it will constitute itself as a valuable diagnosis tool.

## Figures and Tables

**Table 1:** Summary of descriptors for the dataset used.

Number of cases	130
Cancer cases	21
No cancer cases	109
Average age	55.2
Number of significative criteria used	17
Nature of data	Qualitative

**Table 2:** Data associated to the patient’s history.

Data	Comment
Age	-
Personal history	It aims to show if the person had/had not previously any cancer type or any cancer-related issue.
Family history	It assesses whether any breast cancer cases existed in the family’s first- or second-degree members.

**Table 3:** Mammogram-related data.

Data	Comment
Masses	In the case any mass is present, then its shape, margins, and density are documented.
Calcifications	In a similar way to masses, in the case of calcifications, their morphology is documented (these are usually grouped into ‘typically benign’ and ‘suspicious morphology’ categories), and their distribution is characterized. It is also indicated whether the calcifications are either primary or associated, that is, if this is a predominant characteristic or, on the contrary, it is associated to other characteristic, thus having a minor entity.
Architectural distortion and asymmetries	In the case of distortion, it is indicated if the characteristic is present and if it is primary or associated. On the other hand, in the case of asymmetries, it is indicated if any is present, and then its type (focal, in development, etc.).
BI-RADS^©^ indicator	The BI-RADS© (Breast Imaging Reporting and Data System) system is nowadays a widely accepted and used diagnosis instrument in the evaluation of breast cancer. It was developed by the American College of Radiology (ACR) [47] with the goal of homogenizing the assessments by providing a standard operation framework for the study of mammogram images through the use of a common vocabulary and a structuration of the evaluation process. Such system is explained in more detail in Section S2.3 of the Supplementary Materials.
Composition	The breast type, i.e., the tissue type, will also be taken into account. As commented in Section S2.3.1 of the Supplementary Materials, as the breast density increases, it is much more complicated to perform its evaluation, potentially hiding mammogram findings, which implies that the diagnosis might be erroneous.

**Table 4:** General data about the Machine Learning algorithms.

**Weighted KNN Model**
Distance: Euclidean distance
Number of nearest neighbors in X used to classify each point: 10
Distance weight: Squared inverse
**Bagged Trees Model**
Ensemble aggregation method: Bag
Number of ensemble learning cycles: 30
Learners: Decision tree
Maximum number of splits: 218

**Table 5:** Data of the patient to be studied input to the application.

**Mass**	
Present/Absent	Absent
Shape	(None)
Margins	(None)
Density	(None)
**Calcifications**	
Present/Absent	Present
Primary/Associated	Associated
Shape	Coarse heterogeneous
Distribution	Segmental
**Asymmetry**	
Present/Absent	Present
Type	Focal
**Architectural Distortion**	
Present/Absent	Absent
Primary/Associated	(None)
**BI-RADS category**	4A
**Breast density**	Scattered
**Other data**	
Age	53
Patient history	No
Family history	Minor

**Table 6:** Diagnosis returned by the system for the 11 developed cases.

	Mass			Calcifications			Asymmetry	Architectural Distortion	BI-RADS Category	Breast Density	Other Data			Hazard Index	System Advice	Real Chart
	Shape	Margin	Density	Type	Shape	Distribution					Age	Patient History	Family History			
1	-	-	-	Associated	Coarse heterogeneous	Segmental	Focal	.	4A	Scattered	53	No	Minor	66.67	Cancer	Cancer
2	Oval	Circumscribed	High	-	-	-	-	-	4B	Heterogeneously	50	N/A	N/A	63.33	Uncertain	No cancer
3	Irregular	Indistinct	High	-	-	-	-	-	4B	Heterogeneously	42	No	None	56.67	No cancer	No cancer
4	Irregular	Spiculated	Equal	Associated	Coarse heterogeneous	Grouped	-	-	4B	Scattered	65	No	None	53.33	No cancer	No cancer
5	-	-	-	Associated	Amorphous	Grouped	Focal	-	4B	Heterogeneously	31	No	None	56.67	No cancer	No cancer
6	-	-	-	Primary	Amorphous	Grouped	-	-	4B	Heterogeneously	49	Yes	N/A	53.33	No cancer	No cancer
7	-	-	-	-	-	-	-	Primary	4C	Scattered	62	Yes	Major	60	Uncertain	No cancer
8	-	-	-	-	-	-	-	Primary	4B	Scattered	58	No	None	56.67	No cancer	No cancer
9	-	-	-	-	-	-	Developing	-	4B	Scattered	64	No	None	50	No cancer	No cancer
10	-	-	-	Primary	Fine pleomomophic	Grouped	-	-	4B	Scattered	58	No	Major	30	No cancer	No cancer
11	Irregular	Indistinct	High	-	-	-	-	-	4B	Heterogeneously	53	No	Major	87.22	Cancer	Cancer

## Data Availability

Not applicable.

## Supplementary Materials

The following supporting information can be downloaded at https://www.mdpi.com/article/10.3390/jpm12020169/s1, Section S1. General aspects of the intelligent decision system; Section S2. Introduction to the theoretical concepts used; Section S2.1. Clinical decision support systems and expert systems; Section S2.2. Data generation and analysis; Section S2.3. BI-RADS^©^; Section S2.3.1. Findings; Section. S2.3.2. BI-RADS^©^ evaluation categories; Section S3. Adaptation of the proposed system to Hevner’s design guidelines within the Information Systems field; Section S4. Guided process for the use of the developed software application; Section S4.1. Data preparation: normalization and balancing; Section S4.2. Determination of latent factors; Section S4.3. The Machine Learning algorithm; Section S5. Guided process for the case study; Section S5.1. Compilation of characteristics and other information of interest, and expert interpretation; Section S5.2. Data processing and interpretation; Section S5.3. Generation of alerts and decision making; Section S6. Discussion of the system’s relevance within the field of study; Table S1. Malignity suspicion level for the mass’ characteristics—Adapted from Malagelada [47]; Table S2. Malignity suspicion level for calcifications according to distribution—Adapted from Sickles et al. [45]; Table S3. BI-RADS© evaluation categories—Adapted from Sickles et al. [45]; Table S4. Studies carried out and acceptance range; Table S5. Data of the patient to be studied input to the application; Table S6. Comparison results; Figure S1. Dialog box for the normalization and balancing of the starting data; Figure S2. Dialog box for data validation prior to applying factorial analysis; Figure S3. Dialog box of the application for performing the factorial analysis; Figure S4. Module for applying the Machine Learning algorithm; Figure S5. Module for the generation of alerts and decision making; Figure S6. Screen capture of the risk results obtained from the cascaded expert systems; Figure S7. Screen capture of the dataset normalization and balancing module; Figure S8. Screen capture of the exploratory factorial analysis module; Figure S9. Screen capture of the model training module; Figure S10. Screen capture of the generation of alerts and decision-making module.
