# Supplementary material for: Design and Development of an Intelligent Clinical Decision Support System Applied to the Evaluation of Breast Cancer Risk
Source: J Pers Med. 2022 Jan 27;12(2):169. doi: 10.3390/jpm12020169 (PMC8880667; doi:10.3390/jpm12020169)
Supplement: Supplementary file 1 [file jpm-12-00169-s001.zip › jpm-1493465-supplementary.pdf]

Journal of Personalized Medicine

MDPI

Supplementary Materials

Section S1. General aspects of the intelligent decision system.

In the main manuscript, the design and development of a new intelligent clinical decision-support system is presented that provides support in the diagnosis process of breast cancer cases. Starting with the information and the characteristics extracted by the medical professionals from the interpretation of the mammogram images, as well as considering other data of interest about the patient (such as her clinical history) the system provides a quantitative index value of the hazard associated to the potential presence of cancer, from which interpretation it will be possible to determine the diagnosis and the evolution of the treatment. Thus, according to the hazard estimation, some additional tests (other additional image tests, biopsy, blood tests, etc.) might be recommended which, in case of a high hazard level, will confirm the presence of a cancer case, or else will establish a series of routine examinations to continue monitoring the patient's health status. To this end, and aiming to build a support system having higher diagnosis objectivity and lower diagnosis uncertainty levels, the use of an intelligent system is proposed that integrates symbolic artificial intelligence models, represented by the use of expert systems, and statistical inference computational models [1,2], through the use of classification non-parametric inference algorithms that are commonly used in Machine Learning.

The operation of the proposed system will in essence be sequential and consequent. First, the starting dataset will be processed in a compartmented way by means of a series of expert systems, deployed in a cascade, and based on Mamdani-type fuzzy-logic inference engines[3–6]. Their subsequent answers will allow obtaining a series or risk indices associated to the presence of cancer that are inferred from the different data groups. As the risk indices associated to each patient's information are obtained, these are structured and stored together with the 'cancer' or 'non-cancer' label that was previously confirmed in the registration of the starting data. This new database will be then used to train classification algorithms, and later to elaborate a plausible prediction derived from the data on a new patient. It is precisely because of those algorithms and their implicit need for training that it is necessary to label the risk data considering a supervised-learning model.

In the case of having an unbalanced and asymmetric dataset, that is, one having highly biased distributions regarding one of the classes or labels, it will be necessary to artificially normalize and modify the data using normalization algorithms and data-augmentation models. Once the database is created, exploratory factorial analysis techniques will be applied to detect the potential subjacent relationships that are present among the different risk indicators, thus determining these latent factors derived from the starting data that group together and represent them. By transforming the values of the different previously obtained risks, after being normalized and augmented, to the new factors space, and contemplating the labels that they had previously assigned, it is possible to have available a robust dataset to train a non-parametric classification algorithm that allows helping to assess the hazard related to the potential presence of breast cancer in a specific patient, which will allow to propose recommendations. With that, it is achieved to expressly reduce the indetermination in the evaluations, to control its subjectivity and to restrict the uncertainty associated both to the measurements and to the evaluations themselves. With the goal of testing and verifying the proposed system, its implementation has been carried out on the MATLAB© (R2021a, MathWorks©, Natick, MA, USA) software platform.

Section S2. Introduction to the theoretical concepts used.

Aiming to the clarification and extension of the explanation provided about the different theoretical concepts that are part of the decision intelligent system that is introduced in the main manuscript, in this section some of their key aspects are shown in more detail, carrying out a revision of their main concepts and applications.

Section S2.1. Clinical decision support systems and expert systems

Clinical Decision Support Systems (CDSS) aim to help and provide support to decision-making processes in medical-healthcare environments by means of the processing, handling, and representation of information. When applied to the diagnosis, it has been shown the diagnosis improvement that they incorporate, as well as the increase in the quality and satisfaction of the service provided to patients [7–9]. On the light of this definition, it is possible to consider the CDSS as a type of decision support system (DSS) that is specifically applied to the clinical area.

Decision support systems are represented by means of a collection of methodologies and tools that make up a specific research discipline within information systems, because of their versatility of use and their capabilities for adapting and representing data [10–12]. They can be applied in multi-disciplinary environments, in this case, for example, in the healthcare area to help in breast cancer diagnosis, providing an efficient support to the decision-making process whatever the source of the information to be considered might be. Integrating different techniques and approaches, among which the data science and artificial intelligence disciplines might be highlighted, these systems are aimed, not only toward improving the handling and management of information, but also toward driving and improving the decision-making process to guarantee optimal choices.

Expert systems, being the most paradigmatic of the artificial intelligence symbolic models, stand out among all these techniques and approaches having a complementary relationship with DSS. Developed in the 1960 decade [13–15] their use is nowadays common in the fields of decision making and knowledge management. They may be framed within the intelligent systems category [16,17] and considered as computational systems that have the capability for representing and simulating human expertise and knowledge [15,18,19] in a way that they are capable to solve complex problems. Because of the benefits and capabilities of expert systems, these are frequently used in the decision theory field, complementing, and strengthening the DSS [20–26], their presence being common in many study fields such as the medical-healthcare one to which this work is aimed [27].

From the viewpoint of its architecture, for the definition of an expert system it is necessary to have available, in its essential conceptualization, the data of the problem from a real domain, with an expert knowledge base that precisely represents the human knowledge and expertise, a human-machine interface, and an inference engine to determine the solution [13,14,18,19,28–30]. Its own architecture establishes the main classification of expert systems based on their capabilities to handle uncertainty. These systems, working with information, are exposed to the vagueness and inaccuracy that are inherent to the handling and transmission of that information. The control of these imperfections has been widely studied, and it has caused several divisions in the expert systems category. In this way, all those that do not handle uncertainty (or deterministic) are associated to rule-based systems, while those other that do efficiently manage it are associated to stochastic systems, either with probabilistic or non-probabilistic approaches.

It is unquestionable that an expert system applied to decision-making in the sanitary environment must take into account the control of uncertainty, and because of that in this work the use of fuzzy logic-based non-probabilistic inference engines is posed.

Taking into account all that previously mentioned and considering that the development of the clinical decision support system is made within the information systems field, as it will need to be integrated into one of them, it is necessary to consider the proposals by Hevner et al. in their works [31,32], relative to the basics and fundamentals of design science research. From them, it is possible to evaluate the development of

software artifacts within such field, as it is the case of the clinical decision support intelligent system that is proposed in this work. Because of that, the guidelines proposed by Hevner et al. for the evaluation of the design of the proposed artifact will be considered, as argued in Section S3.

Section S2.2. Data generation and analysis

All medical-healthcare applications must start with the premise of having available a statistically coherent and balanced database, that is, without anomalies present, these understood as elements in the set having a low probability of being there [33]. However, on some occasions the generation of unbalanced datasets results to be entirely unavoidable because of the nature of the data to be collected and the different unbalance types that exist [34]. Thus, for example, in two-class datasets, such as the one that will be used in the case study, it is common the presence of a majority dominant more probable class, and a minority less probable class that is usually processed by predictive classification algorithms as noise, producing low-reliability predictions [33–35]. Different solutions exist [35] oriented to the prevention of the potential issues associated to unbalanced data sets. Among all these solutions, those focused on the pre-processing of the starting data have become more relevant in these last years. They are based in performing intentional modifications on the database aiming to balancing the classes by means of the artificial data elimination or addition, using techniques respectively known as sub-sampling and over-sampling, even resorting to hybrid combined solutions of them [34]. Among the different over-sampling alternatives, one of the most popular and accepted ones is Safe-Level SMOTE [36], a variation of SMOTE [37]. The SMOTE technique adds new samples to the minority class in a random way, placing them somewhere in the line connecting the minority class and its nearest neighbor, ignoring which are the minority classes in its environment. This behavior might not be appropriate, and it could cause a fictional over-generation that produces class overlapping, adding noise, and not reflecting the reality. To correct those effects, SMOTE has evolved in an adaptive way to models such as Borderline-SMOTE or Safe-Level SMOTE. In the later, a coefficient named 'safe level' is used that allows to determine which zones are the most convenient to produce synthetic data. SMOTE and its adaptive variants have been used with notable success as over-sampling methods to correct unbalanced data situations in medical-healthcare regression and classification problems [38–40]. In this line, it has been reported that when working with medical datasets [38] it is recommended the combined use of the Z-score normalization technique as a previous step to the performance of the over-sampling using the SMOTE variants.

In parallel to the data generation, and prior to the determination of whether the patient's data show a high danger of suffering breast cancer, it is necessary to find those subjacent factors, non-directly measurable, that might be related to dependency groups based on variance patterns that are shared among the variables representing the risks obtained in the expert systems cascade and the latent factors that are present in the data. To do that, one of the more used and well-known approaches is the Exploratory Factorial Analysis (EFA). In this specific case, because of its popularity and applicability to the case, a choice will be made for the Common Factor Analysis, an approach aimed to express the variance shared by a certain number of variables as a function of a particular set of subjacent factors, so that this reduced number of dimensions succeeds in explaining the most possible information [41,42]. It is clear that, in this example, the goal is not only reducing the dimensionality of the risk data set, in which case a principal component analysis approach would be more convenient, but to determine parameters representing the latent factors themselves, observing and categorizing the covariance among the variables and their load and commonality with the different factors. It is precisely the need for determining and interpreting the relationships that exist between the observed variables and the latent factors from a conceptual viewpoint, determining the logic of these relationships as a future source of diagnosis information, what justifies the election of that method [41,43].

Section S2.3. BI-RADS©

The BI-RADS© (Breast Imaging Reporting and Data System) system is nowadays a widely accepted and used diagnosis instrument in the evaluation of breast cancer. It was developed by the American College of Radiology (ACR) [44] with the goal of homogenizing the assessments by providing a standard operation framework for the study of mammogram images through the use of a common vocabulary and a structuration of the evaluation process. The indications made in the 5th edition of BI-RADS© [44,45] have been considered for the elaboration of this work.

Section S2.3.1. Findings

According to the BI-RADS guidelines, the main types of findings and descriptors that can be detected on a mammography image are the presence of masses, calcifications, architectural mammary distortion, presence of asymmetries, revision of the intra-mammary lymphatic ganglia, presence of skin wounds, presence of a single dilated conduct, location of the lesion, as well as other meaningful findings [45]. Taking into account the criteria used by the specialists that built the previously mentioned dataset, in this work the study will focus specifically on masses, calcifications, architectural distortion and asymmetries, as these are the signs with which breast cancer is usually associated according to the criteria referred before:

- Masses: These are tri-dimensional elements that may be visualized by combining two different mammogram images. According to the 5th BI-RADS© edition, to characterize a mass its shape (oval, rounded or irregular), its margins (circumscribed, darkened, micro-lobulated, undefined or spiculated) and its density (hyper-dense, iso-dense, hypo-dense, or adipose content) will be taken into account [45]. In the current literature there are different works from which it is possible to find criteria that allows to interpret the findings. For example, in the study by Woods et al. [46] it was proved that a high density in masses implies a high malignity risk. As a summary, Table S1 shows the degree of malignity suspicion associated to each one of the elements from the different mass descriptors. It is relevant to mention that, as the used dataset was built between the years 2006 and 2011, thus before the publication of the current BI-RADS© edition, Table S1 incorporates some terms in the 'Shape' columns that are nowadays obsolete.

Table S1. Malignity suspicion level for the mass' characteristics – Adapted from Malagelada [47].

Shape		Margins		Density	Malignity suspicion level
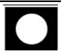	Rounded	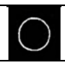	Circumscribed	Adipose	-
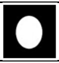	Oval	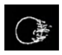	Darkened	Hypo-dense	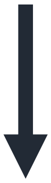
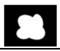	Lobulated	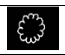	Micro-lobulated		
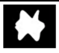	Irregular	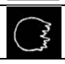	Undefined	Iso-dense	
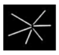	Architectural distorsion	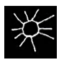	Spiculated	Hyper-dense	+

- Calcifications: These may be classified as: typically benign (skin, vascular, gross or macro-calcified –popcorn-like, thick linear, rounded, annular, dystrophic, calcic-milk or suture-threads), with a suspicious morphology (amorphous, heterogeneous-gross, thin-pleomorphic, and thin-linear or thin-branched-linear), or distribution-related (diffuse, regional, grouped, linear or segmented) [45]. In relation to the interpretation of the malignity suspicion degree of calcifications, the two first groups are clearly characterized. Table S2 shows the different distributions, ordered according to the malignity suspicion level.

Table S2. Malignity suspicion level for calcifications according to distribution – Adapted from Sickles et al. [45].

Distribution	Malignity suspicion level
Diffuse<br>Regional<br>Grouped<br>Linear<br>Segmented	-<br>↓<br>+

- Architectural distortion: This is an alteration of the normal mammary architecture caused by a non-visible and non-touchable mass which is commonly not diagnosed in mammogram images, thus causing false negatives [48]. When the patient has no previous (traumatic or surgical) antecedents, the appearance of a distortion may suggest the presence of cancer [45].
- Asymmetries: These can be classified as: global, focal or in development. Global asymmetry is generally considered as a normal variant. With regard to focal asymmetries, it is common that they become considered as masses after their diagnosis evaluation. Asymmetries in development are new focal asymmetries, about 15% of which result in the appearance of malignant wounds [45].

Additionally, the breast composition is taken into account, categorizing breasts having a mostly adipose tissue, breasts having fibro-glandular density disperse sectors, heterogeneously dense breasts, and highly-dense breasts [45]. It is commonly accepted that, as the breast density increases, the process for its evaluation becomes more difficult, with that density being considered as an independent risk factor [49].

Section. S2.3.2. BI-RADS© evaluation categories

After the mammogram image elaboration and analysis, it is required to describe the anomalies detected in that image by means of the system's own scale, that is the BI-RADS© scale [45]. Such scale has a 0 to 6 range, with a value of 0 corresponding to a non-completed study, so more tests are required, while a value of 6 indicates a malignant diagnosis already confirmed by means of a biopsy.

Table S3. BI-RADS© evaluation categories – Adapted from Sickles et al. [45].

BI-RADS© assessment category	Malignancy probability level
0	N/A
1 − 0% malignancy	-<br>↓<br>+
2 − 0% malignancy	
3 − (0.2] % malignancy	
4 − (2.95] % malignancy<br>* 4A − (2.10]% malignancy<br>* 4B − (10.50]% malignancy<br>* 4C − (50.95]% malignancy	
5 − ≥ 95% malignancy	
6	Confirmed cancer

Table S3 shows the different categories, together with a malignancy probability value. It is important to point out that for the case of category 4 three subcategories are taken into account: 4A with (2.10]% malignancy probability, 4B with (10.50]% malignancy probability and 4C with (50.95]% malignancy probability.

Section S3. Adaptation of the proposed system to Hevner's design guidelines within the Information Systems field.

As it was already mentioned in Section S2 of this Supplementary Materials document, the general theoretical framework on which this work is developed is the information systems context, a fact already argued before, and by which the guidelines proposed by Hevner in 2004 [32] are considered next. The performance analysis for the presented system across these seven guidelines allows to size the nature of the contribution within the information systems scope:

- Guideline 1 — Design an artifact: The proposed clinical decision support system, developed across Section 2 of the article, has been implemented into a software artifact which, by using a set of expert systems deployed in cascade, exploratory factorial analysis, data augmentation approaches and classification algorithms, allows to help in an early detection of patients with potential breast cancer cases. To ease the calculations, the artifact has been implemented on the MATLAB© platform, what allows easing the use of the proposed system.
- Guideline 2 — Relevance of the problem: The design and development of a clinical decision support system oriented towards the early detection of breast cancer cases is a relevant contribution to the healthcare field, as already indicated in Section 1 of the article, because of the high impact this cancer involves as it is one of the main causes of death in women. It is important also to highlight that, in line with what has been already commented, a great variability can exist in the diagnosis process depending on the professional in charge of the evaluation, because of which the developed system may achieve a reduction in that variability.
- Guideline 3 — Design evaluation: In Section 3 of the article, a case study is presented that gives an example of the operation of the proposed decision support system.
- Guideline 4 — Contributions to the field of research: The contributions of the proposed decision support system have been described in Section 1 of the article, to be later expanded and detailed in Section 4 of the article and Section S6 of the Supplementary Materials.
- Guideline 5 — Rigor in the research: The proposed system, framed within the information systems and design science contexts, uses concepts from fuzzy inference systems, these being widely supported in current literature because of their proved capabilities to handle uncertainty across decision-making processes. Additionally, it is common practice in current literature to apply factorial analysis and data-augmentation techniques, as previously mentioned in Section S2.2 of the Supplementary Materials, allowing reducing the dimensionality by combining a series of latent factors using coherent and balanced databases.
- Guideline 6 — Design as a search: A revision was carried out in Section 4 of the article and Section 2.1 of the Supplementary Materials of the theoretical and practical contexts of the decision support system proposed in this article.
- Guideline 7 — Communication of the research: In Section 4 of the article and Section S6 of the Supplementary Materials the contributions of this work are presented.

Section S4. Guided process for the use of the developed software application.

In this section, complementing the explanation made in Section 2.2 of the main article, an illustrated exposition is made of the work and operations flows within the developed software application.

Section S4.1. Data preparation: normalization and balancing

The zone of the application oriented to the normalization and balancing of data is shown inside the red frame in Figure S1. First, the dataset derived from the cascaded expert systems will be loaded by clicking on the Load Data button, which already contains the different risks for each one of the patients in the dataset. After that, all the previously loaded risks are normalized, together with those from the patient, clicking on the Apply Zscore button. Next, the system will suggest the user, in the boxes labelled as Add, how many synthetic data should be generated to have the same number of positive and

negative cases, and the user could change this ratio as wished. Additionally, the user must define the number of neighbors in the Number of Neighbors box, so the algorithm can make the calculations. Finally, the user will click on the Safe Level SMOTE button. The green frame in Figure S1 shows the new normalized and balanced dataset, while inside its blue frame the values of the normalized risks are shown for the patient whose case is under study by the medical team.

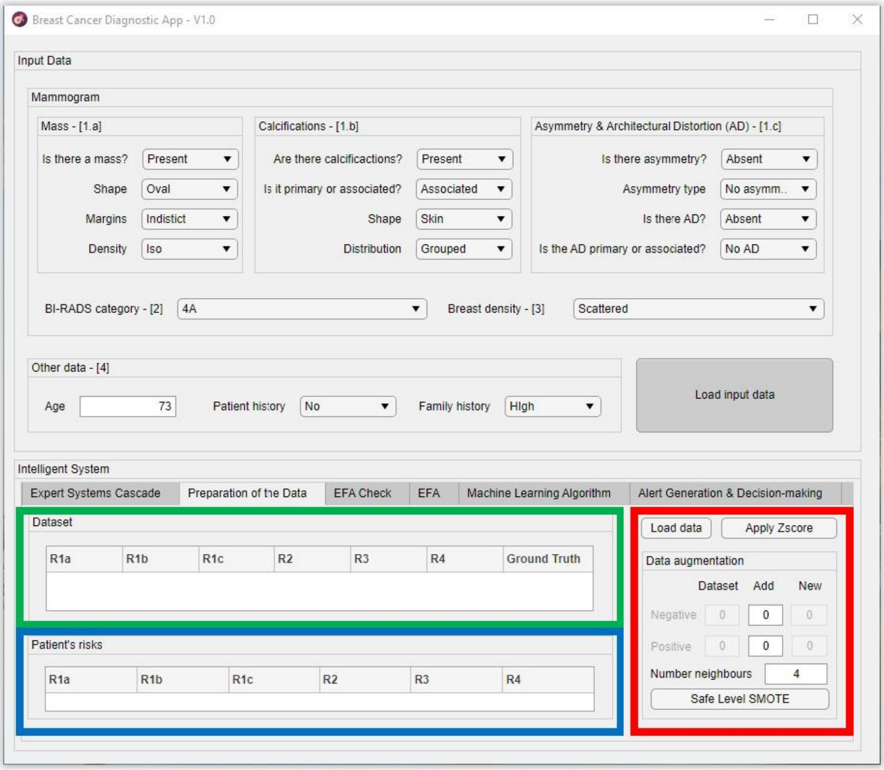

Figure S1. Dialog box for the normalization and balancing of the starting data.

Section S4.2. Determination of Latent Factors

Starting from the data that was normalized and augmented in the previous phase, in this stage those subjacent latent factors that are present in the dataset are determined by using Exploratory Factorial Analysis (EFA) techniques, more specifically the Common Factor Analysis approach. However, as a previous step to the application of EFA, it is necessary to verify the statistical assumptions that are recommended to ensure that the dimensionality reduction process is statistically significant. Said assumptions are often referred, on the one hand to the nature of the data, assessing its normality and multi-collinearity, as well as the presence of atypical data, and on the other hand to the number and interrelation of the data itself. The Shapiro-Wilk test, together with the estimation of the asymmetry indices, will be used for the normality case. The multi-collinearity will search for the presence of strong correlations among data, that are harmful to the analysis; the Variance Inflation Factor (VIF) will be used for that. The Mahalanobis distance will be used for the detection of atypical or marginal data points. Finally, the number of data must be high enough, what is guaranteed by having a ratio of more than 10 data lines per risk (actually, the dataset consists of more than 400 data lines). Regarding data interrelation, this must be guaranteed to exist, because of which Bartlett's Test of Sphericity will be performed, together with the determination of Kaiser-Mayer-Olikin (KMO) index. Table S4 collects all those previously mentioned studies, incorporating an estimation of the values or intervals considered as appropriate.

Table S4. Studies carried out and acceptance range.

**Normality Study**							
Shapiro-Wilk	The Shapiro-Wilk test poses the following hypotheses, in this case, with a significance level of 0.05:<br>- H0: The data present a normal distribution.<br>- H1: The data do not present a normal distribution.<br>After performing the test on the different variables, p-values lower than 0.05 have been obtained, because of which the null hypothesis can be rejected, confirming that the data do not follow a normal distribution.						
Asymmetry Indices	When the symmetry indices are within the ± 1.5 interval, then it may be claimed that small variations are present with respect to the normal distribution. All the variables in the dataset present symmetry indices contained into that interval, except for R1c that shows a value of 2.301.						
**Multi-Collinearity Study**	**C_{ref}**	**R_{1a}**	**R_{1b}**	**R_{1c}**	**R_2**	**R_3**	**R_4**
Variance Inflation Factor (VIF)	VIF > 10 indicates high col-linearity	2.593	2.29	2.643	4.291	3.789	1.728
**Atypical data study**							
Mahalanobis Dis-tance	The calculation of Mahalanobis distance has been performed on the generated database. After that, a cut distance is determined using the Chi-square inverse cumulative distribution function for 6 degrees of freedom and a probability of 0.999, that is, for a sig-nification level p<0.001.<br>After performing the calculation, it was observed the presence of three potentially atypical data lines which, after being reviewed, it was decided to keep them.						
**Data Interrelation Study**							
Bartlett's Test of Sphericity	The following hypotheses are posed in Bartlett's Test of Spheric-ity, in this case with a signification level of 0.05:<br>- H0: The correlations matrix is an identity matrix (there is no correlation).<br>- H1: The correlations matrix is not an identity matrix (there is correlation).<br>A p-value=0 is obtained, so the null hypothesis must be rejected, and it may be claimed that the variables are correlated enough to apply EFA.						
Kaiser-Mayer-Olikin (KMO) Index	The KMO index is in the range 0-1, and as the index value grows, more correlated the data are. Values above 0.5 might be accepta-ble to perform EFA.<br>In this case a value of 0.467 is obtained, because of which the data is appropriate to perform EFA.						

In Figure S2 a tab is incorporated, named *EFA Check*, where the assessment indices values of the prior statistical assumptions of EFA can be verified.

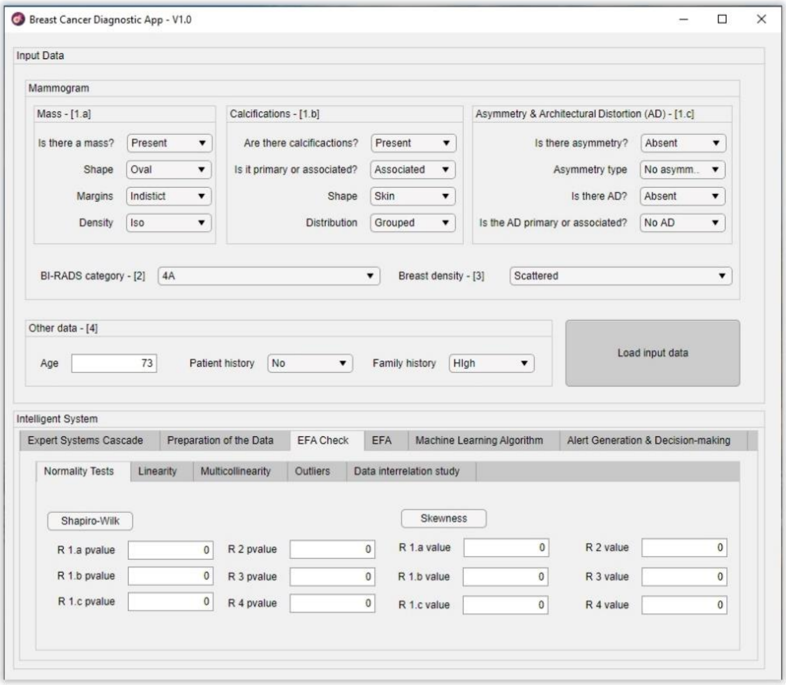

Figure S2. Dialog box for data validation prior to applying factorial analysis.

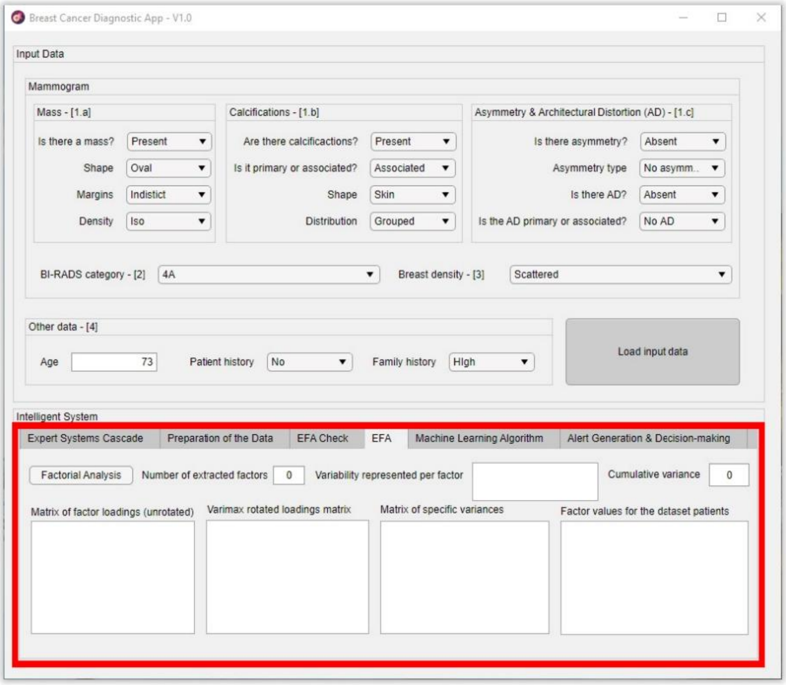

Figure S3. Dialog box of the application for performing the factorial analysis.

Figure S3 shows the dialog box used for performing the factorial analysis. The user must only click on the *Factorial Analysis* button and the calculation process will be automatically carried out, obtaining the number of extracted factors, as well as the value of each factor for each one of the starting datasets. Additionally, several additional data related to the calculation process are shown. The first frame shows the factorial loads matrix. After this box, the loads matrix already rotated using Varimax is shown. The third box presents the specific variances matrix. The last box, in the line of the already mentioned, provides the values of the factors for each patient in the dataset, derived from the factorial score obtained after the application of the Anderson-Rubin method, chosen here because Varimax was used in the rotation. The factorial score allows characterizing each

patient in each one of the determined factors, providing a qualitative metric of the quality in dimensionality reduction and in the explanation of the involved information.

Section S4.3. The Machine Learning algorithm

In Figure S4, the red frame highlights the panel for choosing and training the classification algorithm. Additionally, a window is presented in which the user can observe the ROC curve for the model. Also, a table is provided on the right of the graph, with data of interest about the different points in the ROC curve (data associated to the confusion matrix, sensitivity, and specificity), that may help the user to determine which operation point is to be selected.

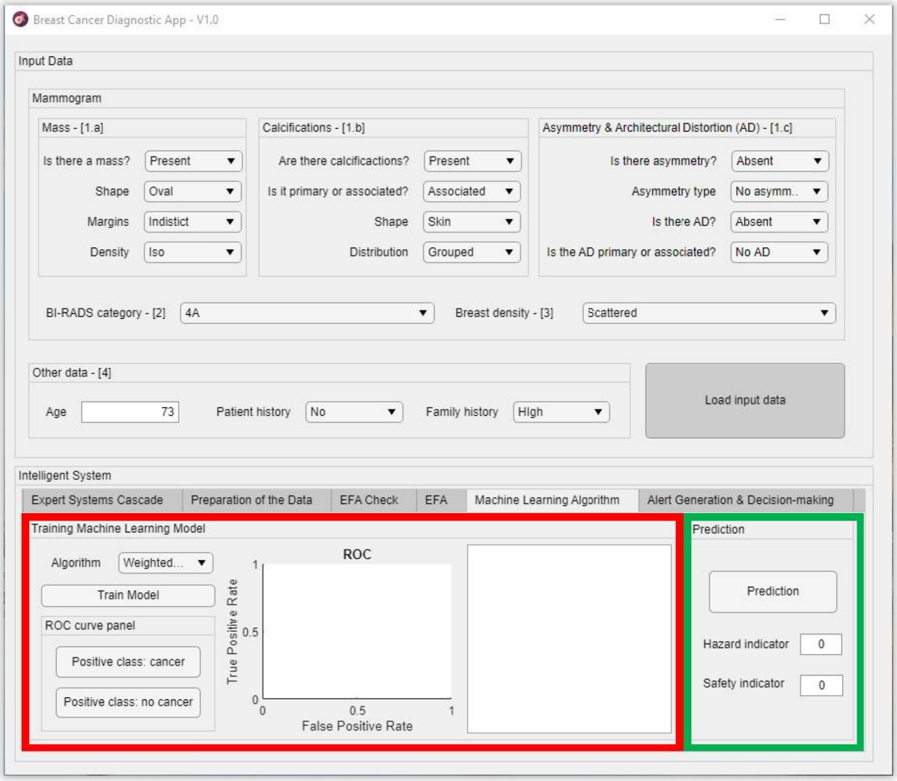

Figure S4. Module for applying the Machine Learning algorithm.

In the green frame of Figure S4, it is performed the calculation of the scores derived from the factor values for the patient case, on the one hand that associated to the patient having cancer, and on the other hand to the patient not having cancer, these being respectively named for the sake of this work 'Hazard index' and 'Safety index'. The Hazard index may be understood as a danger index associated to the patient potentially having cancer, while the Safety index can be interpreted as a safety index associated to the patient potentially not having cancer. These factors are within the 0-1 range, and they must add up to one. To facilitate their use, in this work they will be scaled to a 0-100 range. From the interpretation of these scores and taking into account the ROC curve for the used model, it will be possible to determine the patient's status.

Section S4.4. Generation of alerts & decision making

Figure S5 shows a screen capture of the application in which it is possible, first to define the operation limits for the system, and after that to determine the system's recommendation. Furthermore, that recommendation comes with a luminous signal showing green, orange, and red colors for status 1, 2 and 3, respectively, aiming to indicate the danger level that each patient has.

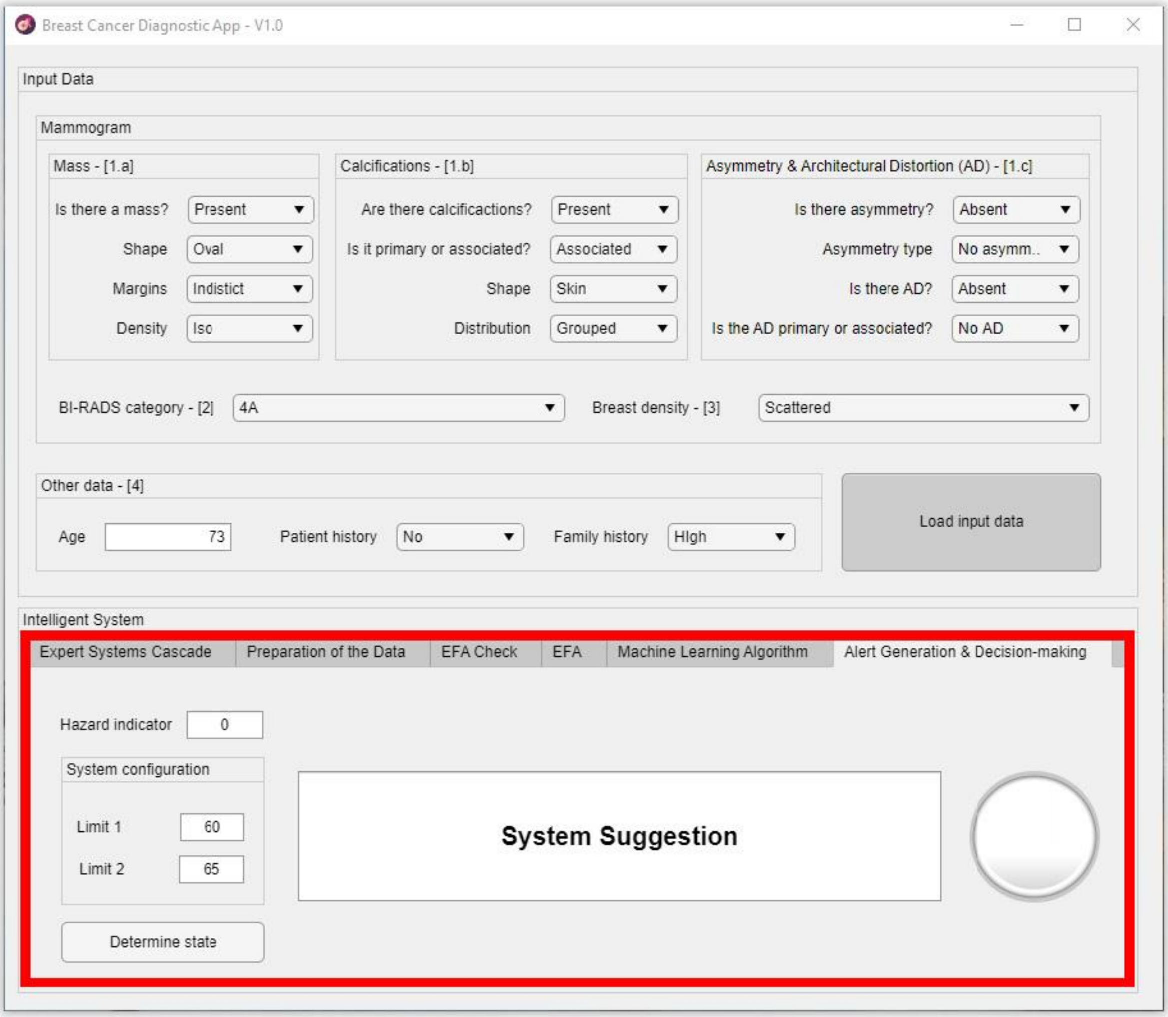

Figure S5. Module for the generation of alerts and decision-making.

Section S5. Guided process for the case study.

This section complements what was explained in the Results section of the main article. In it, an illustrated revision of the process for using the application for the diagnosis of a typical patient will be shown. Aiming to facilitate its understanding, some of the texts are directly replicated from the main manuscript.

Section S5.1. Compilation of characteristics and other information of interest, and expert interpretation

As already mentioned, the presented system starts its operation with the data being input by the professional into the application. Table S5 shows a summary of the patient's data to be analyzed in this case study She is a 53-year old patient, with a family history of low cancer risk, without having previously debuted in cancer. After evaluating her mammogram, it is possible to observe the presence of associated calcifications, with a coarse heterogeneous shape and a segmental distribution. A focal asymmetry was also observed. It is relevant to highlight that this patient was diagnosed with cancer. All those data, compiled in Table S5, have been introduced into the application, in the *Input Data* box of Figure S6.

Table S5. Data of the patient to be studied input to the application.

**Mass**	
Present/Absent	Absent
Shape	(None)
Margins	(None)
Density	(None)
**Calcifications**	
Present/Absent	Present
Primary/Associated	Associated
Shape	Coarse heterogeneous
Distribution	Segmental
**Asymmetry**	
Present/Absent	Present
Type	Focal
**Architectural Distortion**	
Present/Absent	Absent
Primary/Associated	(None)
**BI-RADS category**	4A
**Breast density**	Scattered
**Other data**	
Age	53
Patient history	No
Family history	Minor

Section S5.2. Data processing and interpretation

After inputting the data into the application, it is possible to proceed to its processing by the system.

First, the risks calculation is carried out in the cascaded expert systems, as previously mentioned in Section 2.2.1 of the article, with the results shown in Figure S6 inside the *Expert System Cascade* module.

The risk value associated to the masses, R1a, shows a value of 0.01538, the risk value associated to the calcifications, R1b, shows a value of 39.97, the risk value associated to asymmetries and architectural distortion, R1c, shows a value of 70.03, and finally the risk value associated to the BI-RADS© indicator and to the first-level risks, R2, shows a value of 89.98. On the other hand, the risk value associated to the breast density and to the risk values of the first and second levels, R3, shows a value of 80. Finally, the risk value associated to age, patient/family history and to the first, second and third levels, R4, shows a value of 89.97.

As it can be observed, in this case the higher risk values are those associated to the architectural distortion & asymmetries, to the BI-RADS, to the breast density and to the patient's age/history, that is, the R1C, R2, R3 and R4 risks.

Once the risk values for the patient to be studied have been calculated, it is proceeded to load the training dataset, to perform their normalization, and to apply Safe-Level SMOTE (a clear asymmetry is observed in it), adding 189 samples for the 'cancer' class and 100 for the 'non-cancer' one, which makes the training dataset to have almost the same number of 'cancer' and 'non-cancer' classes, presenting more than 400 data lines. The normalization of the previously calculated risks for the patient to be studied is also performed. All that procedure is illustrated in Figure S7.

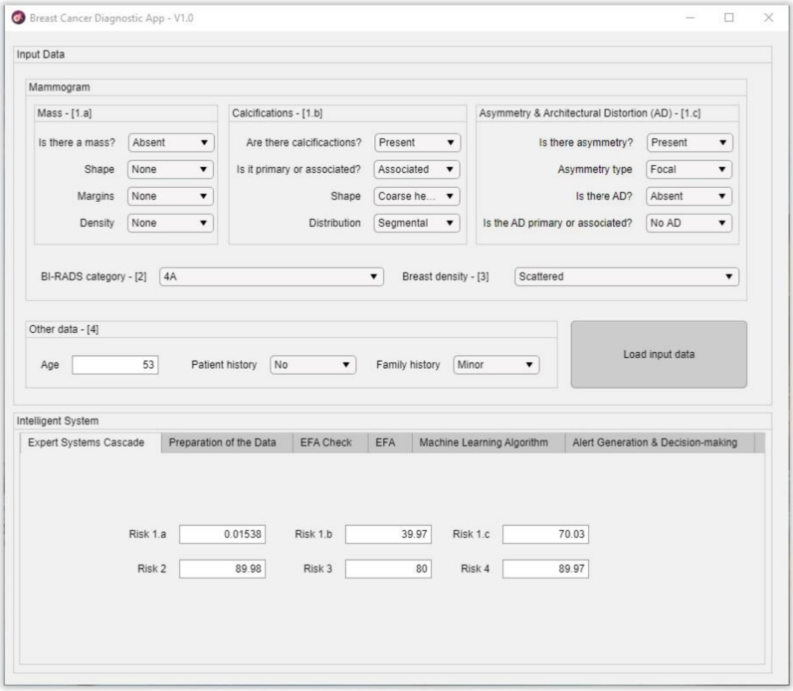

Figure S6. Screen capture of the risk results obtained from the cascaded expert systems.

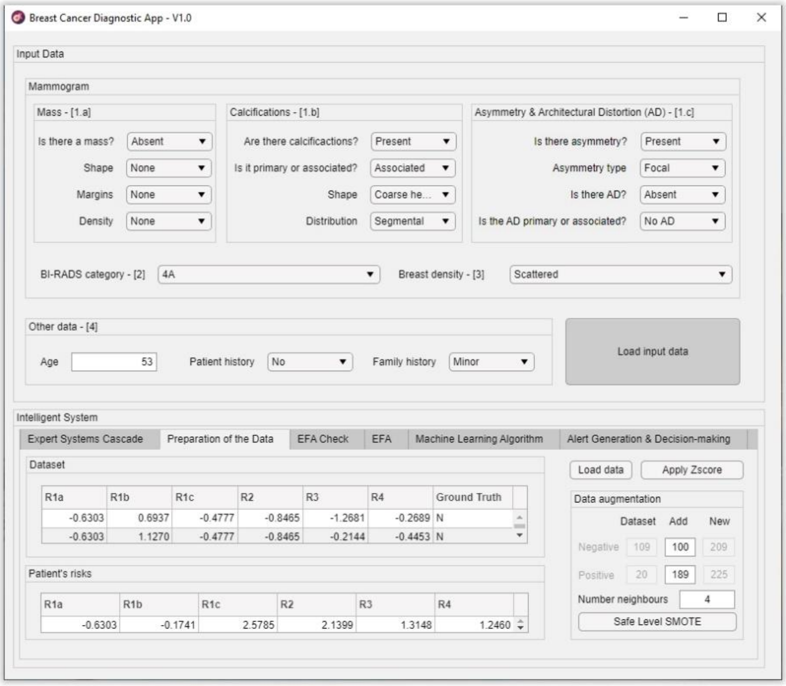

Figure S7. Screen capture of the dataset normalization and balancing module.

Taking the augmented dataset, exploratory factorial analysis techniques are applied next after verifying its pertinence, as previously described in Section 2.2.3 of the main article, and as shown in Figure S8. In line with that already explained, three latent factors are extracted which, on this dataset, allow to explain a 75.28% of the total variability.

For this case:

- Factor 1, the one presenting larger rotated loads for R2, R3 y R4, represents a variability value of 36.81% for the starting data.
- Factor 2, the one having larger rotated loads for R1a and R1b, represents a variability value of 21.45% for the starting data.

- Factor 3, the one associated with R1c because it presents a larger load, represents a variability value of 17.03% for the starting data.

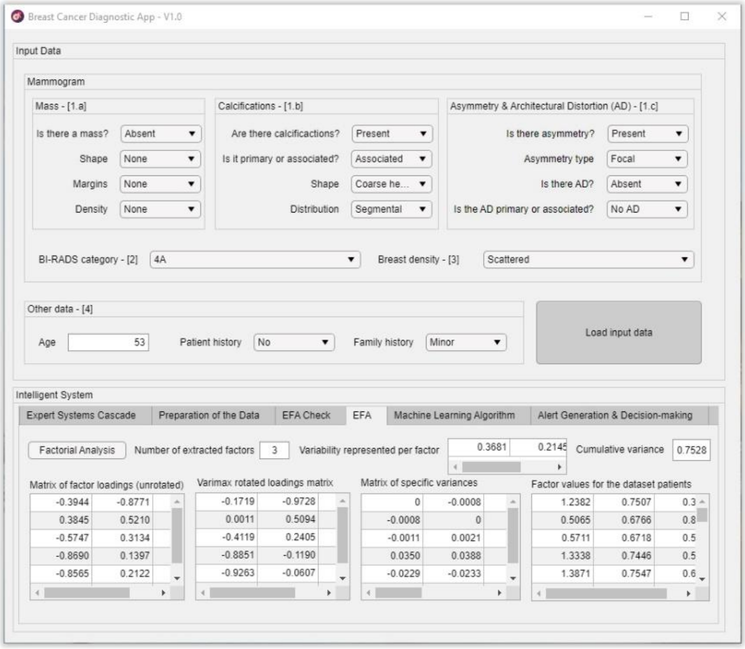

Figure S8. Screen capture of the exploratory factorial analysis module.

The starting values (the risks) have been also mapped to the new factors space, using the Anderson-Rubin factorial scoring method, both for the dataset and for the data of the patient to be analyzed.

Once this is done, it is proceeded to train the Machine Learning model, using Bagged Tree in this case, as described in Section 2.2.4 of the article and as it can be seen in Figure S9. The scores for the patient's data are also calculated, obtaining a Hazard index value of 66.67.

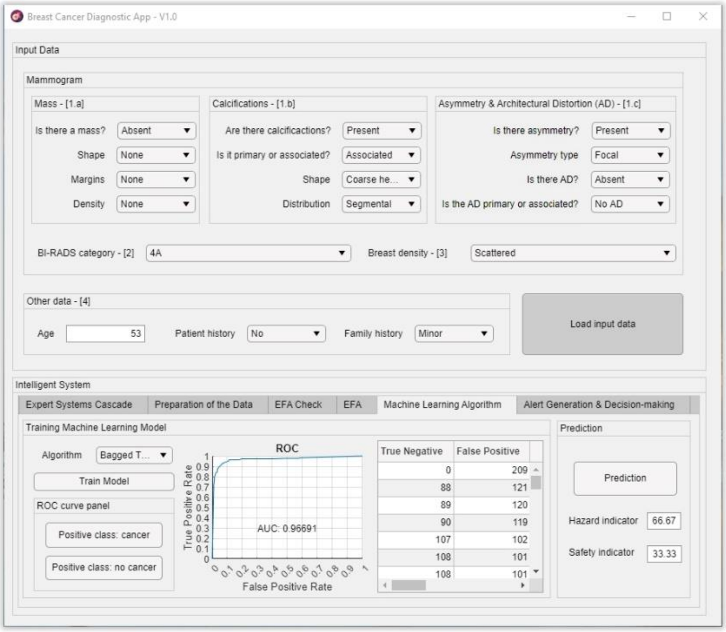

Figure S9. Screen capture of the model training module.

Section S5.3. Generation of alerts and decision-making

After the model has been trained and the scores for the patient to be studied have been calculated, it is possible to proceed to the generation of alerts and the decision-making process.

Taking into account limit values of 60 and 65, established after analyzing the ROC curve, in this specific case the system is facing a potential cancer case, in which it is recommended to the medical-healthcare professionals to perform more tests, starting from the least aggressive ones, that could help to make a diagnosis decision, determining whether it will be finally necessary to proceed to biopsy tests. Figure S10 shows the module associated with this stage.

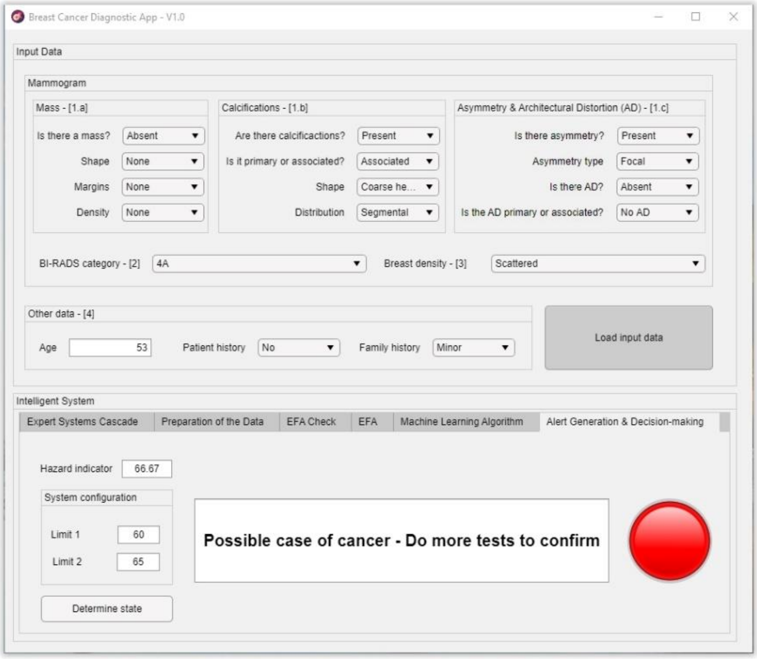

Figure S10. Screen capture of the generation of alerts and decision-making module.

Section S6. Discussion of the system's relevance within the field of study.

The use of clinical decision support systems is common in medical-healthcare environments, either integrated into information systems or as standalone tools for a local or particular use [7]. These systems incorporate many tools that range from the simple data processing to those other having learning and inference capabilities [14,50,51]. In this last group, it is common the incorporation of expert systems, as well as the use of factorial analysis (exploratory and/or confirmatory), and of Machine Learning itself. However, the conjoint, compensated, and justified use of all these approaches within a necessarily intelligent system is an unquestionable novelty. The 'intelligent system' concept, derived from artificial intelligence, acts in this work as an aggregator of methods that use different knowledge representations. Nevertheless, all of them efficiently complement one another and perform consequent representation and inferences that increase the relevance of the final result. The posed intelligent system possesses, therefore, perception, reasoning, learning and inference capabilities [16]. In the same way, among other features of the intelligent systems [17], the one presented in this work is able to reason logically, solve complex problems, can be adapted to different scenarios and makes an effective use of the existing information. Thus, it is unquestionable the intelligent nature of the system that, additionally, is reached by means of the integrated models. The combined operation of these models, in turn, presents notable challenges that have been solved in this work. The data significance and relationships have not been altered across the processing chain, and the incorporation of deductive reasoning, typical of expert systems, has been complemented by the more inductive approach that is typical of EFA. Uncertainty, in all its

meanings, has been (directly or indirectly) controlled in the models, and the logic that is present in the medical diagnosis base has been respected and reinforced. The case study shows these results, as well as the system's applicability and robustness. At this moment, the authors may conclude that the incorporation of a different dataset would not return lower success rate values than those obtained in the case study. In this point, it is difficult to perform a relevant comparison of the presented method to other methods and approaches that are used in breast cancer diagnosis, as none of them poses an aggregated intelligent system. Notwithstanding, Table S6 shows a formal comparison of different models found in literature to the current intelligent system, according to the five following criteria: efficiency (understood as the reliability of the diagnosis results considering uncertainty management), scalability (as a feature related to the incorporation of elimination of calculation elements to/from the system), inference (related to the system's capability for symbolic, inductive and deductive reasoning), learning (associated to the capability to learn and incorporate knowledge), and finally adaptability (understood as the capability for adapting to the diagnosis of other diseases).

Table S6. Comparison results.

Method/System	Efficiency	Scalability	Inference	Learning	Adaptability
Ferreira et al. [52]	An inductive logical programming (ILP) approach is used that does not manage uncertainty, so its efficiency is limited.	The system consists only of a single inductive logical programing system. It is not scalable.	The system uses an inductive symbolic reasoning method.	The system focuses in knowledge modelling, so it has moderate capabilities to incorporate new knowledge.	The ILP system used might be adapted to the diagnosis of other diseases, by using other starting data in a different study domain.
	-	-	=	-	=
Côrte-Real et al. [53]	A probabilistic inductive logical programming (PILP) approach is used. Uncertainty is managed by using a probabilistic approach.	The system consists only of a probabilistic inductive logical programming system. It is not scalable.	The system uses an inductive symbolic reasoning method.	The system focuses on knowledge modelling, so it has moderate capabilities to incorporate new knowledge.	The PILP system use might be adapted to the diagnosis of other diseases, by using other starting dada in a different study domain.
	=	-	=	-	=
Alaa et al. [54]	A classifier is proposed that implicitly manages uncertainty.	The system consists of a clustering module followed by a supervised learning algorithm, a classifier in this case. It is scalable.	Statistical inference is used instead of symbolic reasoning.	The system incorporates knowledge in a way that is subsidiary to its classification process.	The system might be adapted to other pathologies.
	-	=	-	-	=
Jiang et al. [55]	Bayesian networks are used, with implicit uncertainty management based on probabilistic calculations.	The system is not scalable, as it is associated to the network model .	Statistical inference is used instead of symbolic reasoning.	The system incorporates knowledge in a way that is subsidiary to the Bayesian network.	The system might be adapted to the recommedation or treatments for other diseases.
	=	-	-	-	=
Abou et al. [56]	Fuzzy logic-based programming approaches are used, so it manages uncertainty by means of a non-probabilistic ap-	The system is not scalable, as it is exclusively associated to the inference model.	The system uses a deductive symbolic reasoning method.	The system possesses a knowledge base associated to the inference engine. It has the capability to incorporate new	The system might be used for the diagnosis of other diseases.

	proach.			knowledge.	
	=	-	=	=	=
Fernandes et al. [57]	The proposed system uses a decision trees-inspired classifier that does not manage uncertainty.	The system is not scalable.	The system uses statistical inference instead of symbolic reasoning.	The system incorporates knowledge in a way that is subsidiary to its classification process.	The system could not be easily used for the diagnosis of other diseases.
	-	-	-	-	-
Proposed system	The proposed system manages uncertainty by means of the use of both probabilistic and non-probabilistic approaches.	The system is scalable. It is possible to modify, incorporate or eliminate calculation and inference blocks.	The system uses deductive symbolic reasoning methods and statistical inference models.	The system has capabilities to model and incorporate new knowledge, and to learn across the process.	The system can be quickly adapted to the diagnosis of other diseases.

It is possible to observe in the previous table that the introduced system, besides being the only one that might be characterized as intelligent, satisfactorily fulfills all the indicated criteria. The results that are pointed out for all the previously mentioned works do not achieve the success rates of the current system, which does not lessen their validity, but it invites to consider the usefulness of the indicated intelligent system. All that involves a meaningful starting point, with wide growth possibilities in the field of clinical decision support systems.

Section S7. References

1. Casella, G.; Berger, R.L. *Statistical Inference*; Duxbury Thomson Learning: Pacific Grove, CA, USA, 2002;
2. D. R. Cox *Principles of Statistical Inference*; Cambridge university press: New York, New York, USA, 2006; ISBN 9780511813559.
3. Mamdani, E.H. Advances in the Linguistic Synthesis of Fuzzy Controllers. *International Journal of Man-Machine Studies* **1976**, *8*, 669–678, doi:10.1016/S0020-7373(76)80028-4.
4. Mamdani, E.H. Application of Fuzzy Logic to Approximate Reasoning Using Linguistic Synthesis. *IEEE Transactions on Computers* **1977**, *C–26*, 1182–1191, doi:10.1109/TC.1977.1674779.
5. Mamdani, E.H.; Assilian, S. An Experiment in Linguistic Synthesis with a Fuzzy Logic Controller. *International Journal of Man-Machine Studies* **1975**, *7*, 1–13, doi:10.1016/S0020-7373(75)80002-2.
6. Ross, T.J. *Fuzzy Logic with Engineering Applications: Third Edition*; Third edit.; John Wiley & Sons, Ltd: Chichester, UK, 2010; ISBN 9781119994374.
7. *Clinical Decision Support Systems*; Berner, E.S., Ed.; Health Informatics; Springer International Publishing: Cham, 2016; ISBN 978-3-319-31911-7.
8. Bright, T.J.; Wong, A.; Dhurjati, R.; Bristow, E.; Bastian, L.; Coeytaux, R.R.; Samsa, G.; Hasselblad, V.; Williams, J.W.; Musty, M.D.; et al. Effect of Clinical Decision-Support Systems: A Systematic Review. *Annals of Internal Medicine* **2012**, *157*, 29–43, doi:10.7326/0003-4819-157-1-201207030-00450.
9. Kawamoto, K.; Houlihan, C.A.; Balas, E.A.; Lobach, D.F. Improving Clinical Practice Using Clinical Decision Support Systems: A Systematic Review of Trials to Identify Features Critical to Success. *British Medical Journal* **2005**, *330*, 765–768, doi:10.1136/bmj.38398.500764.8f.
10. Burstein, F.; W. Holsapple, C. *Handbook on Decision Support Systems 1*; Springer: Berlin, Germany, 2008;
11. Burstein, F.; W. Holsapple, C. *Handbook on Decision Support Systems 2*; Springer: Berlin, Germany, 2008;
12. Bonczek, R.H.; Holsapple, C.W.; Whinston, A.B.; Carter, H. *Foundations of Decision Support Systems*; Academic Press: New York, NY, USA, 1981;

13. Lucas, P.J.F.; van der Gaag, L.C. *Principles of Expert Systems*; Addison-Wesley: Wokingham, UK, 1991; ISBN 0-201-41640-9.
14. Krishnamoorthy, C.S.; Rajeev, S. *Artificial Intelligence and Expert Systems for Engineers*; CRC Press: Boca Raton, FL, USA, 1996;
15. Liao, S.H. Expert System Methodologies and Applications-a Decade Review from 1995 to 2004. *Expert Systems with Applications* **2005**, *28*, 93–103, doi:10.1016/j.eswa.2004.08.003.
16. Karray, F. o.; De Silva, C. *Soft Computing and Intelligent Systems Design*; Pearson Education: Essex, England, 2004; ISBN 9780126464900.
17. Bielawski, L.; Lewand, R. *Intelligent Systems Design: Integrating Expert Systems, Hypermedia, and Database Technologies*; John Wiley & Sons: New York, NY, USA, 1991; ISBN 0471525359.
18. Kumar, Y.; Jain, Y. Research Aspects of Expert System. *International Journal of Computing & Business Research* **2012**, *1*, 1–11.
19. Merritt, D. *Building Expert Systems in Prolog*; Springer: New York, NY, USA, 1989; ISBN 978-1-4613-8913-2.
20. Marston, M.; Allen, J.; Mistree, F. The Decision Support Problem Technique: Integrating Descriptive and Normative Approaches in Decision Based Design. *Engineering Valuation and Cost Analysis* **2000**.
21. Allen, J.K.; Seepersad, C.C.; Choi, H.-J.; Panchal, J.H.; McDowell, D.L.; Mistree, F. Decision Making in Engineering Design. In *Integrated Design of Multiscale, Multifunctional Materials and Products*; Elsevier: Burlington, MA, USA, 2010.
22. Mistree, F.; Smith, W.F.; Bras, B.; Allen, J.K.; Muster, D. Decision-Based Design: A Contemporary Paradigm for Ship Design. *Transactions, Society of Naval Architects and Marine Engineers* **1990**, *98*, 565–597.
23. Chien, C.F.; Kim, K.H.; Liu, B.; Gen, M. Advanced Decision and Intelligence Technologies for Manufacturing and Logistics. *Journal of Intelligent Manufacturing* 2012, *23*, 2133–2135.
24. Hazelrigg, G.A. A Framework for Decision-Based Engineering Design. *Journal of Mechanical Design* **1998**, doi:10.1115/1.2829328.
25. Lewis, K.; Chen, W.; Schmidt, L.; Chen, W. Decision Making in Engineering Design; Asme Press: New York, NY, USA 2006.
26. Pfeifer, R.; Lüthi, H.-J. Decision Support Systems And Expert Systems: A Complementary Relationship? In *Expert Systems and Artificial Intelligence in Decision Support Systems*; Springer Netherlands, 1987; pp. 41–51.
27. Thornett, A.M. Computer Decision Support Systems in General Practice. *International Journal of Information Management* **2001**, doi:10.1016/S0268-4012(00)00049-9.
28. Myers, W. Introduction to Expert Systems. *IEEE Expert* **1986**, *1*, 100–109, doi:10.1109/MEX.1986.5006506.
29. Buchanan, B.G. Expert Systems: Working Systems and the Research Literature. *Expert Systems* **1986**, *3*, 32–50, doi:10.1111/j.1468-0394.1986.tb00192.x.
30. Todd, B.S. *An Introduction to Expert Systems; Oxford University Computing Laboratory*; Oxford, UK, 1992;
31. Hevner, A.R.; Chatterjee, S. *Design Research in Information Systems: Theory and Practice*; Springer: New York, NY, USA, 2010; ISBN 978-1-4419-6107-5.
32. Hevner, A.R.; March, S.T.; Park, J.; Ram, S. Design Science in Information Systems Research. *MIS Quarterly: Management Information Systems* **2004**, *28*, 75–105, doi:10.2307/25148625.
33. Haixiang, G.; Yijing, L.; Shang, J.; Mingyun, G.; Yuanyue, H.; Bing, G. Learning from Class-Imbalanced Data: Review of Methods and Applications. *Expert Systems with Applications* **2017**, *73*, 220–239, doi:10.1016/J.ESWA.2016.12.035.
34. He, H.; Garcia, E.A. Learning from Imbalanced Data. *IEEE Transactions on Knowledge and Data Engineering* **2009**, *21*, 1263–1284, doi:10.1109/TKDE.2008.239.

35. Longadge, R.; Dongre, S. Class Imbalance Problem in Data Mining Review. *International Journal of Computer Science and Network* **2013**, *2*.
36. Bunkhumpornpat, C.; Sinapiromsaran, K.; Lursinsap, C. Safe-Level-SMOTE: Safe-Level-Synthetic Minority over-Sampling Technique for Handling the Class Imbalanced Problem. In Proceedings of the Lecture Notes in Computer Science (including subseries Lecture Notes in Artificial Intelligence and Lecture Notes in Bioinformatics); Springer, Berlin, Heidelberg, 2009; Vol. 5476 LNAI, pp. 475–482.
37. Chawla, N. v.; Bowyer, K.W.; Hall, L.O.; Kegelmeyer, W.P. SMOTE: Synthetic Minority over-Sampling Technique. *Journal of Artificial Intelligence Research* **2002**, *16*, 321–357, doi:10.1613/jair.953.
38. Mohammed, A.J.; Hassan, M.M.; Kadir, D.H. Improving Classification Performance for a Novel Imbalanced Medical Dataset Using Smote Method. *International Journal of Advanced Trends in Computer Science and Engineering* **2020**, *9*, 3161–3172, doi:10.30534/ijatcse/2020/104932020.
39. Abdoh, S.F.; Abo Rizka, M.; Maghraby, F.A. Cervical Cancer Diagnosis Using Random Forest Classifier with SMOTE and FEAture Reduction Techniques. *IEEE Access* **2018**, *6*, 59475–59485, doi:10.1109/ACCESS.2018.2874063.
40. Razzaghi, T.; Safro, I.; Ewing, J.; Sadrfaridpour, E.; Scott, J.D. Predictive Models for Bariatric Surgery Risks with Imbalanced Medical Datasets. *Annals of Operations Research 2019 280:1* **2019**, *280*, 1–18, doi:10.1007/S10479-019-03156-8.
41. Widaman, K.F. Common Factor Analysis Versus Principal Component Analysis: Differential Bias in Representing Model Parameters? *Multivariate Behavioral Research* **1993**, *28*, 263–311, doi:10.1207/s15327906mbr2803_1.
42. Yang, B. Factor Analysis Methods. In *Research in organization. Foundations and Methods of Inquiry.*; Swanson, R.A., Elwood F. Holton, Eds.; Berrett-Koehler Publishers, Inc.: San Francisco, 2005.
43. Velicer, W.F.; Jackson, D.N. Component Analysis versus Common Factor Analysis: Some Issues in Selecting an Appropriate Procedure. *Multivariate Behavioral Research* **1990**, *25*, 1–28, doi:10.1207/s15327906mbr2501_1.
44. Breast Imaging Reporting & Data System | American College of Radiology Available online: https://www.acr.org/Clinical-Resources/Reporting-and-Data-Systems/Bi-Rads (accessed on 28 May 2021).
45. Sickles, E.A.; D'Orsi, C.J.; Bassett, L.W.; Appleton, C.M.; Berg, W.A.; Burnside, E.S.; Feig, S.A.; Gavenonis, S.C.; Newell, M.S.; Trinh, M.M. BI-RADS Del ACR: Mamografía. In *Atlas BI-RADS del ACR, Sistema normalizado para la redacción de informes de estudios por imágenes de la mama*; Colegio Estadounidense de Radiología: Reston, VA, 2013.
46. Woods, R.W.; Sisney, G.S.; Salkowski, L.R.; Shinki, K.; Lin, Y.; Burnside, E.S. The Mammographic Density of a Mass Is a Significant Predictor of Breast Cancer. *Radiology* **2011**, *258*, 417–425, doi:10.1148/radiol.10100328.
47. Malagelada, A.O. *Automatic Mass Segmentation in Mammographic Images - PhD Thesis*; Girona, 2007; ISBN 9788469080191.
48. Pinochet Tejos, M.Á.; Altamirano Salazar, A.V.; Horvath Polos, E.; Uchida Silva, M.; Silva Fuente-Alba, C.; Darrás Ismael, C. Distorsión de La Arquitectura Mamaria: La Mejor Forma de Enfrentarla. *Revista Chilena de Radiologia* **2016**, *22*, 158–163, doi:10.1016/j.rchira.2016.11.007.
49. Hruska, C.B.; Weinmann, A.L.; Tello Skjerseth, C.M.; Wagenaar, E.M.; Conners, A.L.; Tortorelli, C.L.; Maxwell, R.W.; Rhodes, D.J.; O'Connor, M.K. Proof of Concept for Low-Dose Molecular Breast Imaging with a Dual-Head CZT Gamma Camera. Part II. Evaluation in Patients. *Medical Physics* 2012, *39*, 3476–3483.
50. Turban, E.; Aronson, J.E.; Liang, T.-P. *Decision Support Systems And Intelligent Systems, 7th Ed.*; 2007; ISBN 9780470755891.
51. Herrmann, Jeffrey.W. *Engineering Decision Making and Risk Management*; John Wiley & Sons, Inc.: New Jersey, NJ, USA, 2015; ISBN 978-1-118-91933-0.

52. Ferreira, P.; Dutra, I.; Salvini, R.; Burnside, E. Interpretable Models to Predict Breast Cancer. In Proceedings of the 2016 IEEE International Conference on Bioinformatics and Biomedicine, BIBM 2016; Institute of Electrical and Electronics Engineers Inc., January 17 2017; pp. 1507–1511.
53. Côrte-Real, J.; Dutra, I.; Rocha, R. On Applying Probabilistic Logic Programming to Breast Cancer Data. In Proceedings of the Inductive Logic Programming; Lachiche, N., Vrain, C., Eds.; Springer International Publishing: Cham, 2018; pp. 31–45.
54. Alaa, A.M.; Moon, K.H.; Hsu, W.; Van Der Schaar, M. ConfidentCare: A Clinical Decision Support System for Personalized Breast Cancer Screening. *IEEE Transactions on Multimedia* **2016**, *18*, 1942–1955, doi:10.1109/TMM.2016.2589160.
55. Jiang, X.; Wells, A.; Brufsky, A.; Neapolitan, R. A Clinical Decision Support System Learned from Data to Personalize Treatment Recommendations towards Preventing Breast Cancer Metastasis. *PLoS ONE* **2019**, *14*, e0213292, doi:10.1371/journal.pone.0213292.
56. Abou, A.; Ebrahim, S.; Awad, A. A Fuzzy Decision Support System for Management of Breast Cancer. *International Journal of Advanced Computer Science and Applications* **2011**, *2*, doi:10.14569/ijacsa.2011.020307.
57. Fernandes, A.S.; Alves, P.; Jarman, I.H.; Etchells, T.A.; Fonseca, J.M.; Lisboa, P.J.G. A Clinical Decision Support System for Breast Cancer Patients. *IFIP Advances in Information and Communication Technology* **2010**, *314*, 122–129, doi:10.1007/978-3-642-11628-5_13.
